# Supplementary material for: Physical activity during pregnancy and offspring cardiovascular risk factors: findings from a prospective cohort study
Source: BMJ Open. 2013 Sep 25;3(9):e003574. doi: 10.1136/bmjopen-2013-003574 (PMC3787488; doi:10.1136/bmjopen-2013-003574)
Supplement: Author's manuscript [file bmjopen-2013-003574.draft_revisions.pdf]

Physical activity during pregnancy and offspring cardiovascular risk factors: findings from a prospective cohort study

Journal:	*BMJ Open*
Manuscript ID:	bmjopen-2013-003574
Article Type:	Research
Date Submitted by the Author:	09-Jul-2013
Complete List of Authors:	Millard, Louise; University of Bristol, School of Social and Community Medicine<br>Lawlor, Debbie; Department of Social Medicine, University of Bristol, Department of Social Medicine<br>Fraser, Abigail; University of Bristol, School of Social and Community Medicine<br>Howe, Laura; University of Bristol, Social Medicine
**Primary Subject Heading**:	Epidemiology
Secondary Subject Heading:	Paediatrics
Keywords:	Paediatric cardiology < PAEDIATRICS, PAEDIATRICS, EPIDEMIOLOGY

SCHOLARONE™
Manuscripts

Physical activity during pregnancy and offspring cardiovascular risk factors: findings from a prospective cohort study

Louise A C Millard, MSc[1,2*], Debbie A Lawlor, PhD [1,2], Abigail Fraser, PhD [1,2], Laura D Howe, PhD[1,2]

1 – MRC Integrative Epidemiology Unit at the University of Bristol,
2 – School of Social and Community Medicine, University of Bristol

*Corresponding author.
Correspondence to: LAC Millard, School of Social and Community Medicine, University of Bristol, Oakfield House, Bristol, UK, BS8 2BN, [louise.millard@bristol.ac.uk], tel +44 117 331 0084, fax: +44 117 331 0123

For peer review only

For peer review only - http://bmjopen.bmj.com/site/about/guidelines.xhtml

ABSTRACT

Objectives

The long term consequences of maternal physical activity during pregnancy for offspring cardiovascular health are unknown. We examined the association of maternal self-reported physical activity in pregnancy (18 weeks gestation) with offspring cardiovascular risk factors at age 15.

Design

Prospective cohort study.

Setting

The Avon Longitudinal Study of Parents and Children (ALSPAC).

Participants

4,665 maternal-offspring pairs (based on a sample with multivariate multiple imputation to deal with missing data) from the Avon Longitudinal Study of Parents and Children, a prospective cohort based in the South West of England with mothers recruited in pregnancy in 1991-1992.

Primary and secondary outcome measures

Offspring cardiovascular risk factors at age 15; body mass index, waist circumference, systolic blood pressure, diastolic blood pressure, glucose, insulin, low density lipoprotein cholesterol, high density lipoprotein cholesterol and triglycerides.

Results

Greater maternal physical activity was associated with lower BMI, waist circumference, glucose and insulin in unadjusted analyses. The magnitude of associations was generally small with wide confidence intervals, and most associations attenuated towards the null after adjusting for confounders. The strongest evidence of association after adjustment for confounders was for glucose; a one standard deviation greater physical activity during pregnancy was associated with a -0.013 mmol/l difference in offspring glucose levels (equivalent to approximately one third of a standard deviation; 95% confidence interval -0.027 to 0.001 mmol/l).

For peer review only - http://bmjopen.bmj.com/site/about/guidelines.xhtml

Conclusions

Our results suggest that maternal physical activity in pregnancy is unlikely to be an important determinant of later offspring cardiovascular health. There was some suggestion of association with offspring glucose, but given that all other associations (including insulin) were null after adjustment for confounders, this result should be interpreted with caution.

For peer review only

For peer review only - http://bmjopen.bmj.com/site/about/guidelines.xhtml

ARTICLE SUMMARY

Article focus

- To examine the association of maternal self-reported physical activity in pregnancy (18 weeks gestation) with offspring cardiovascular risk factors at age 15.

Key messages

- Physical activity in pregnancy is unlikely to be an important determinant of later offspring cardiovascular health.

Strengths and limitations of this study

- The main strengths of our study are the large sample size, prospective nature of data collection, and the detailed measurement of a wide range of offspring cardiovascular risk factors in adolescence.
- A limitation of our work is that the data on maternal physical activity during pregnancy were self-reported, and thus measurement error is possible. We cannot assess women's physical activity levels before pregnancy, or the extent to which women changed their physical activity levels between the 18 week questionnaire and the end of the pregnancy. Thus it is possible that physical activity later in pregnancy may be a more important exposure for offspring cardiovascular risk.

For peer review only

INTRODUCTION

There is increasing interest in the potential effects of physical activity during pregnancy on both mother and offspring [1]. Current policy encourages physical activity during pregnancy, with UK guidelines recommending at least 30 minutes of moderate intensity activity at least 4 times a week [2-4], and US guidelines recommending 2 ½ hours each week [5]. However, the evidence regarding potential risks and benefits of physical activity during pregnancy is unclear, with a Cochrane systematic review of randomised controlled trials concluding that regular aerobic exercise during pregnancy (mostly swimming, static cycling, floor exercises) improves women's physical fitness, but that there was insufficient evidence to draw firm conclusions about any other short- or long-term risks or benefits to the woman or her offspring [1].

Physical activity is associated with lower cardiovascular risk in both adults and children [6-8]. It is possible that greater levels of physical activity during pregnancy might improve later offspring cardiovascular health, either through direct intrauterine mechanisms influencing musculoskeletal and other system development or because mothers who are more active in pregnancy are also likely to be so postnatally and might encourage their children to be more active [9-12]. There is some evidence that greater levels of physical activity during pregnancy is associated with later offspring growth and lower adiposity [13-16]. All but one of these studies [16] had a very small sample size, and none were able to assess other (than adiposity) cardiovascular risk factors in offspring. Furthermore, a recent study [17] found evidence of an adverse effect of physical activity measured at 30 weeks gestation on high density lipoprotein cholesterol, diastolic blood pressure and body mass index (BMI). Thus, it is unclear whether variation in levels of physical activity during pregnancy is (either beneficially or detrimentally) related to long-term cardiovascular health in offspring.

The aim of this study is to investigate the association of physical activity during pregnancy with a range of offspring cardiovascular risk factors (BMI, waist circumference, glucose, insulin, lipids, and blood pressure). We further aim to explore whether any observed associations are explained by measured confounders, or if not, whether they are mediated by intrauterine characteristics such as hypertensive disorders of pregnancy (HDP), gestational diabetes or gestational weight gain, or whether they are mediated by the offspring's own later physical activity levels.

For peer review only - http://bmjopen.bmj.com/site/about/guidelines.xhtml

METHODS

The Avon Longitudinal Study of Parents and Children (ALSPAC) is a prospective, population-based study investigating environmental and other factors that affect the health and development of children. The study methods are described in detail elsewhere [18 19] and on the study website (**http://www.alspac.bris.ac.uk**). In brief, all pregnant women living in the three health districts centered around Bristol, in the South West of England, who had an expected delivery date between April 1, 1991 and December 31, 1992 were invited to take part in the study. Ethics approval for the study was obtained from the ALSPAC Law and Ethics Committee and the Local Research Ethics Committee.

From the 14,062 live births in the study, we restricted analyses to singleton pregnancies of offspring alive at 1 year, and excluded 1,234 women who did not provide data on physical activity during pregnancy, 1 woman with an implausible value of physical activity during pregnancy and 7,063 offspring without data for at least one outcome, yielding a sample size of 4,665 mother-offspring pairs (see Fig. 1 for the study participant flow diagram).

Physical activity during pregnancy

At 18 weeks gestation, women were asked to report the hours per week that they currently spend doing each of 11 types of leisure time physical activities (brisk walking, swimming, antenatal exercise, keep fit exercise, cycling, aerobics, tennis, yoga, jogging, weight training, squash, other exercise). Based on the responses to these questions, we estimated a weighted activity index (i.e. the weekly total energy expenditure from these activities) by multiplying the published average metabolic equivalent (MET) scores for the reported activities [20] with the estimated hours per week (7 for ‘‘≥7 h’’, 4 for ‘‘2–6 h’’, 0.5 for ‘‘<1 h’’, and 0 for ‘‘never’’) – see Supplemental Table 1 (Online Resource) for example MET scores. Published MET values are not available for ‘antenatal exercise’ and ‘keep-fit exercise’, thus we assigned 2.0 and 3.5 METs, respectively. Physical activity equating to a score greater than 70 was reported by 46 individuals, which is equivalent to 7 hours per week of jogging; these values were re-coded to 70 in order to prevent them from having undue influence on the results [21].

Cardiovascular risk factors in the offspring

Offspring cardiovascular risk factors (body mass index (BMI), waist circumference, glucose, insulin, lipids, and blood pressure) were measured at a research clinic attended at mean age 15.5 years (further details are provided

For peer review only - http://bmjopen.bmj.com/site/about/guidelines.xhtml

in Online Resource).

Other measurements

We considered the following to be potential confounding factors: household social class, maternal education, ethnicity, parity (the number of previous pregnancies resulting in a live birth or stillbirth), smoking during pregnancy, previous history of hypertension outside of pregnancy, pre-pregnancy BMI, and gestational weight gain in the first 18 weeks of pregnancy. We also adjusted for the offspring's gender and age at assessment of outcomes in all analyses.

We considered the following to be potential mediating factors: HDP, gestational weight gain (between 18-28 weeks and 28 weeks onwards; see Online Resource for explanation of why these two time points were used), gestational diabetes, offspring gestational age, birthweight and offspring accelerometer-assessed moderate to vigorous physical activity at 14 years. Gestational age and birthweight are plausible mediators of the intrauterine pathways due to their association with both gestational diabetes and HDP, and offspring outcomes [10 11]. Details of the assessment of all of these potential confounders and mediators are provided in Online Resource.

Statistical Analysis

All analysis was performed with Stata v11.2 [22]. Insulin and triglycerides were positively skewed and so were log transformed. Multivariable linear regression was used to examine the association between the weighted score of maternal physical activity during pregnancy and offspring cardiovascular risk factors. The weighted score of maternal physical activity in pregnancy was standardized to have a mean of zero and variance of one. Coefficients from regression models therefore represent the mean difference in cardiovascular risk factors (in their measured units or a percentage difference for the log transformed insulin and triglyceride outcomes) for a one standard deviation increase in MET physical activity, which is equivalent to approximately 15 MET hours per week, i.e. just over two hours of jogging, or just under four hours of brisk walking. We also repeated all analyses with each cardiovascular outcome scaled per standard deviation of their distribution (or logged distribution for insulin and triglycerides) and present these results in figures. This allowed us to compare the magnitudes of any associations across outcomes. We used tests of interaction to assess whether the associations between maternal physical activity during pregnancy and offspring cardiovascular risk factors differed by child gender or by maternal pre-pregnancy BMI (categorized as underweight/normal weight [BMI less than 25] or

For peer review only - http://bmjopen.bmj.com/site/about/guidelines.xhtml

overweight/obese [BMI 25 or above]) [23].

Fig. 2 illustrates the hypothesised pathways between maternal physical activity during pregnancy and offspring cardiovascular risk factors. We carried out several analyses to explore these pathways. We initially investigated minimally adjusted associations, adjusting only for the child's gender and exact age at outcome measurement. Confounder-adjusted models adjusted for child gender, exact age at outcome measurement, household social class, maternal education, ethnicity, parity, smoking during pregnancy, previous hypertension, pre-pregnancy weight and gestational weight gain between 0-18 weeks gestation. The possible mediating mechanisms involved were investigated in two further models: a) all confounders plus intrauterine characteristics – HDP, gestational diabetes and gestational weight gain after 18 weeks, gestational age and birthweight, and b) all confounders plus offspring physical activity at age 14. A final model included all potential confounding and mediating factors.

Missing data

We used multivariate multiple imputation (details in Online Resource) to impute missing data for all mother-offspring pairs where the data on maternal physical activity during pregnancy (the exposure) and at least one cardiovascular risk factor at age 15 was observed (the eligible sample, N=4,665). Analyses conducted using this imputation dataset were considered our main analyses. However, in order to assess the impact of missing data on our results, we compared the results of the main analysis (using imputed datasets) with results from a complete case approach in which we included only individuals with complete data for all outcomes, all confounders and all mediators. Characteristics of participants and levels of missing data for each variable are shown in Supplementary Table 2 (Online Resource).

For peer review only

For peer review only - http://bmjopen.bmj.com/site/about/guidelines.xhtml

RESULTS

There was large inter-individual variation in reported MET-hours per week at 18 weeks gestation. Approximately one third of mothers reported less than 6 MET hours per week, which equates to one hour of swimming, whilst approximately one quarter of women reported over 24 MET hours per week. On average, higher social class, greater maternal education, lower parity, no maternal smoking in pregnancy and lower maternal pre-pregnancy BMI were associated with greater reported physical activity at 18 weeks gestation (Table 1). There was limited evidence that maternal physical activity was associated with most of our a priori specified mediators of the association between physical activity in pregnancy and offspring cardiovascular risk factors; no strong evidence of association was seen for hypertensive disorders of pregnancy, diabetes, gestational weight gain, gestational age, or birth weight, but greater maternal physical activity during pregnancy was associated with greater offspring physical activity at age 14. For each standard deviation increase in reported physical activity during pregnancy, there was a 0.06 SD increase in physical activity in the offspring (95% CI 0.03 to 0.09) (Supplementary Table 3 in Online Resource).

Association between physical activity during pregnancy and offspring cardiovascular risk factors

There was no evidence that the associations between maternal physical activity during pregnancy and offspring cardiovascular risk factors differed either by offspring gender (all *P* for interaction > 0.2) or by maternal pre-pregnancy BMI status (all *P* for interaction > 0.1 see Supplemental Figure 1 in Online Resource). Fig. 3 and Table 2 show the minimally adjusted, confounder adjusted and mediator (intrauterine characteristics and offspring physical activity) adjusted associations of maternal physical activity during pregnancy with offspring cardiovascular risk factors. More detailed results from models examining each potential mediator separately are given in Supplemental Table 4 in Online Resource).

There was little evidence that maternal physical activity during pregnancy was associated with the offspring cardiovascular risk factors. In minimally adjusted analyses (adjusting only for child gender and age at outcome assessment), higher levels of physical activity were associated with lower offspring BMI, smaller waist circumference, lower glucose, and lower insulin. For all outcomes except insulin and glucose, the magnitude of coefficients was small and confidence intervals were wide. A one standard deviation increase in physical activity during pregnancy is associated with a lower insulin level in the offspring of -1.90% (95% CI -3.50 to -0.30) and lower glucose level by -0.013 mmol/l (95% CI -0.027 to 0.001). Adjustment for measured confounders moved

For peer review only - http://bmjopen.bmj.com/site/about/guidelines.xhtml

most of the associations towards the null. After adjusting for confounding, one standard deviation increase in physical activity during pregnancy is associated with lower offspring insulin by -1.20% (95% CI -2.80 to 0.40). The association with glucose did not alter after adjusting for confounders. Adjusting for potential mediators had little effect on the estimated associations for any outcome.

Sensitivity of results to missing data

The results based on multivariate multiple imputation were very similar to those based on individuals with complete data (Supplemental Figure 2 for BMI, Supplemental Figure 3 for insulin and Supplemental Figure 4 for glucose are shown as illustrative examples, in Online Resource).

For peer review only

For peer review only - http://bmjopen.bmj.com/site/about/guidelines.xhtml

DISCUSSION

Despite greater maternal physical activity in pregnancy being associated with lower pre-pregnancy BMI and greater offspring physical activity at mean age 14 years, there was no strong evidence that it was related to later offspring cardiovascular risk factors (BMI, waist circumference, blood pressure (systolic blood pressure, diastolic blood pressure), lipids (triglycerides, low density lipoprotein cholesterol, high density lipoprotein cholesterol), and fasting glucose or insulin) at age 15 years using data from a contemporary general population pregnancy cohort. The associations we observed were small, and were generally attenuated with adjustment for measured confounders. The strongest evidence of association was for glucose (one standard deviation greater physical activity in pregnancy was associated with a lower glucose by -0.013 mmol/l (95% confidence interval -0.027 to 0.001), equivalent to approximately one third of a standard deviation), but given that we investigated several outcomes, that the association with all other outcomes including insulin attenuated towards the null after adjustment for confounding, and that no associations were seen for BMI, waist circumference or any other cardiovascular risk factor, this finding should be interpreted with caution and requires replication in other studies.

To our knowledge only one study to date has investigated the association of maternal physical activity during pregnancy with a range of offspring cardiovascular risk factors [17]. This study included 965 Danish participants at age 20, and examined the associations of self-reported physical activity at work, leisure time physical activity, daily amount of walking or cycling, and sport participation at 30 weeks gestation with offspring cardiovascular risk factors. The authors found that higher levels of self-reported walking-cycling at 30 weeks gestation was associated with higher offspring BMI and diastolic blood pressure, lower high density lipoprotein cholesterol, and (in males only) higher systolic blood pressure and waist circumference. The associations were weak and of small magnitude. No associations were seen for the other aspects of physical activity assessed in the analysis. It is therefore possible that the associations observed in this study occurred due to chance. However, the authors' conclusions are in line with our own, in that there was no evidence to support the a priori hypothesised potential benefit of physical activity during pregnancy for offspring cardiovascular health.

The main strengths of our study are the large sample size, prospective nature of data collection, and the detailed measurement of a wide range of offspring cardiovascular risk factors in adolescence. The measure of maternal physical activity during pregnancy uses a weighted index, which attempts to measure the energy

For peer review only - http://bmjopen.bmj.com/site/about/guidelines.xhtml

expenditure of individuals using previously published average values for different types of physical activity (see Supplemental Table 1 in Online Resource). We were able to assess the extent to which our observed associations are confounded by maternal pre-pregnancy BMI and socio-economic factors, and explore a number of potential a priori specified mediating pathways (intrauterine factors and shared familial environment, represented by the child's own physical activity). Additionally, we used multivariate multiple imputation to examine and correct for potential bias introduced by missing data.

The results using complete case data and using multivariate multiply imputed data were similar. In both analyses, the conclusion remains that there is no strong association between reported levels of physical activity during pregnancy and offspring BMI (or any other cardiovascular risk factor). Multiple imputation gives unbiased results providing the data are missing at random, i.e. assuming that the missingness is not related to the value of the observation, conditional on the variables included in the imputation model. We included in the imputation models a wide range of variables predictive of both missingness in the outcomes and of levels of cardiovascular risk factors in those with observed data, including socio-economic factors, maternal and family characteristics, and earlier measures of the offspring's cardiovascular disease risk factors, in order to make sure that this assumption holds.

A limitation of our work is that the data on maternal physical activity during pregnancy were self-reported, and thus measurement error is possible. However, associations of socioeconomic position, pre-pregnancy BMI, maternal education and objectively measured offspring physical activity at age 14 were as expected, providing some evidence of face validity. We also cannot assess women's physical activity levels before pregnancy, or the extent to which women changed their physical activity levels between the 18 week questionnaire and the end of the pregnancy as these data are not available in this cohort. Thus it is possible that physical activity later in pregnancy may be a more important exposure for offspring cardiovascular risk – further studies are necessary to test this hypothesis.

Although we demonstrated no evidence of association between physical activity during pregnancy and offspring cardiovascular risk factors, we did find that greater levels of physical activity during pregnancy were associated with greater levels of objectively assessed physical activity in the offspring at mean age 14 years. This measure of offspring physical activity has been shown to be associated with fat mass and cardiovascular risk factors [8 24].

Our results, in line with the only existing similar study to date [17], suggest that maternal physical activity in pregnancy is unlikely to be an important determinant of later offspring cardiovascular health, though

For peer review only - http://bmjopen.bmj.com/site/about/guidelines.xhtml

further large prospective studies of this association are needed to be confident that this is the case, particularly with studies using objectively measured physical activity at various times throughout pregnancy.

For peer review only

For peer review only - http://bmjopen.bmj.com/site/about/guidelines.xhtml

FOOTNOTES

Contributors

Louise A C Millard: Ms. Millard contributed to the design of the study, performed the analyses, drafted the initial manuscript, reviewed and revised the manuscript, and approved the final manuscript as submitted. Debbie A Lawlor: Professor Lawlor conceptualised and designed the study, critically reviewed and revised the manuscript, and approved the final manuscript as submitted. Abigail Fraser: Dr Fraser critically reviewed and revised the manuscript, and approved the final manuscript as submitted. Laura D Howe: Dr Howe contributed to the design of the study, supervised the analyses, critically reviewed and revised the manuscript, and approved the final manuscript as submitted.

Funding

LACM is funded by a studentship from the UK Medical Research Council. LDH was funded by a UK Medical Research Council Population Health Scientist Fellowship (G1002375). AF is funded by a UK Medical Research Council research fellowship (grant 0701594). The UK Medical Research Council (grant G074882), the Wellcome Trust (grant WT076467), and the University of Bristol provide core funding support for ALSPAC. The UK Medical Research Council (grant G0600705) and the University of Bristol provide core funding for the Medical Research Council Centre for Causal Analyses in Translational Epidemiology.

Ethics approval

ALSPAC Law and Ethics Committee and the Local Research Ethics Committees.

Data sharing statement

The ALSPAC policy on data sharing is available on the website: http://www.bristol.ac.uk/alspac. To discuss access to ALSPAC data, please contact the ALSPAC executive team on alspac-exec@bristol.ac.uk.

For peer review only

TABLES

Table 1 Association of participant characteristics with maternal physical activity during pregnancy: using multivariate multiply imputed data (N = 4,665) of individuals with data for maternal physical activity (exposure) and at least one outcome

Potential confounder	Confounder category	Standard deviation (SD) change of maternal physical activity during pregnancy associated with a 1 SD increase of confounder, with 95% confidence interval
Household social class [a]	Class I (professional)	Reference
	Class II	0.00 (-0.08 to 0.09)
	Class III non-manual	-0.18 (-0.27 to -0.09)
	Class III manual	-0.16 (-0.28 to -0.04)
	Class IV/V (manual)	-0.15 (-0.31 to 0.01)
Maternal education [b]	Less than O-level	Reference
	O-level	0.17 (0.09 to 0.25)
	A-level	0.39 (0.30 to 0.47)
	Degree or above	0.40 (0.30 to 0.49)
Ethnicity [c]	Non-white	Reference
	White	-0.11 (-0.25 to 0.03)
Parity [d]	0	Reference
	1	-0.05 (-0.11 to 0.02)
	2 +	-0.10 (-0.19 to -0.02)
Maternal smoking in pregnancy	No	Reference
	Yes	-0.09 (-0.17 to -0.01)
Previous hypertension	No	Reference
	Yes	-0.03 (-0.17 to 0.12)
Maternal pre-pregnancy BMI (SD)		-0.39 (-0.58 to -0.20)
Gestational weight gain 0-18 weeks (SD)		-0.00 (-0.03 to 0.03)

[a] Household social class: The mother recorded the occupation of both herself and her partner in a questionnaire at 32 weeks gestation, which were used to allocate them to social class groups using the 1991 Office of Population, Censuses and Surveys classification; the highest class of the mother and her partner was used in analysis
[b] Maternal education: O-level (ordinary level) exams are taken in different subjects usually at age 15–16 at the completion of legally required school attendance, equivalent to today's General Certificate of Secondary Education); A-level (Advanced-level) exams are taken in different subjects usually at age 18)
[c] Ethnicity: Mixed was recoded as non-white due to the small numbers in the mixed category (n=3)
[d] Parity: Obtained from obstetric records. Mothers with parity of two or more were grouped into a single category

For peer review only - http://bmjopen.bmj.com/site/about/guidelines.xhtml

Table 2 Association between maternal physical activity during pregnancy and offspring cardiovascular risk factors: using multivariate multiply imputed data (N = 4,665) of individuals with data for maternal physical activity (exposure) and at least one outcome

	Change per 1 SD greater maternal metabolic equivalent physical activity (95% confidence intervals)		
**Outcome**	**Minimally adjusted**	**Confounder adjusted**	**All confounders and mediators**
**Mean difference (in units specified in first column) per 1 standard deviation (SD) greater maternal physical activity during pregnancy**			
BMI, kg/m^2	-0.089 (-0.190 to 0.011)	0.004 (-0.091 to 0.098)	0.023 (-0.071 to 0.117)
Waist circumference, cm	-0.271 (-0.552 to 0.011)	-0.088 (-0.358 to 0.181)	-0.042 (-0.311 to 0.227)
SBP, mmHg	-0.044 (-0.360 to 0.272)	-0.014 (-0.332 to 0.303)	0.017 (-0.301 to 0.334)
DBP, mmHg	0.015 (-0.251 to 0.280)	0.017 (-0.251 to 0.284)	0.018 (-0.250 to 0.286)
Glucose, mmol/L	-0.013 (-0.027 to 0.001)	-0.013 (-0.027 to 0.001)	-0.012 (-0.026 to 0.001)
LDLc, mmol/L	-0.006 (-0.025 to 0.013)	-0.002 (-0.022 to 0.017)	-0.003 (-0.022 to 0.017)
HDLc, mmol/L	0.002 (-0.008 to 0.012)	0.000 (-0.010 to 0.010)	-0.001 (-0.011 to 0.009)
**Percentage difference per 1 standard deviation (SD) greater maternal physical activity during pregnancy**			
Insulin, % difference	-1.90 (-3.50 to -0.30)	-1.20 (-2.80 to 0.40)	-1.00 (-2.60 to 0.60)
Triglycerides, % difference	0.00 (-1.20 to 1.30)	0.00 (-1.30 to 1.20)	0.00 (-1.20 to 1.40)

Results of additional models with adjustment for mediators are in Supplemental Table 4 in Online Resource

Minimally adjusted: Adjusted for child gender and child age at 15 year clinic

Confounder adjusted: Adjusted for child age at 15 year clinic, child gender, socioeconomic position confounders (household social class, maternal education, ethnicity, parity, mother smoker, previous hypertension), maternal pre-pregnancy BMI (weight & height) and gestational weight gain 0-18 weeks

All confounders and mediators: Confounder adjusted with additional adjustment for intrauterine mechanisms (HDP (none, gestational hypertension, preeclampsia), diabetes/glycosuria (non, existing diabetes, gestational diabetes, glycosuria), gestational weight gain 18-24 weeks, gestational weight gain 24 weeks onwards), birth weight, gestational age, and child's physical activity at age 14

For peer review only - http://bmjopen.bmj.com/site/about/guidelines.xhtml

FIGURES

Fig. 1 Flow chart of ALSPAC study participants

Fig. 2 Pathway illustration for the potential associations between physical activity during pregnancy and offspring cardiovascular risk factors

Fig. 3 Associations of maternal physical activity during pregnancy with offspring cardiovascular risk factors: using multivariate multiply imputed data (N = 4,665) of individuals with data for maternal physical activity (exposure) and at least one outcome

Footnotes for Figure 3:

Mean standard deviation (SD) difference (and 95% confidence intervals) in offspring cardiovascular risk factors (or logged distribution for insulin and triglycerides) for a one SD increase in metabolic equivalent maternal physical activity

Minimally adjusted: Adjusted for child gender and child age at 15 year clinic

Confounder adjusted: Adjusted for child age at 15 year clinic, child gender, socio-economic position confounders (household social class, maternal education, ethnicity, parity, mother smoker, previous hypertension), maternal pre-pregnancy BMI (weight & height) and gestational weight gain 0-18 weeks

Fully adjusted with adjustment for all confounders and mediators: Confounder adjusted with additional adjustment for intrauterine mechanisms (HDP (none, gestational hypertension, preeclampsia), diabetes/glycosuria (non, existing diabetes, gestational diabetes, glycosuria), gestational weight gain 18-24 weeks, gestational weight gain 24 weeks onwards), birth weight, gestational age, and child's physical activity at age 14

For peer review only - http://bmjopen.bmj.com/site/about/guidelines.xhtml

REFERENCES

1. Kramer MS, McDonald SW. Aerobic exercise for women during pregnancy. Cochrane Database Syst Rev 2006;**3**:CD000180 doi: 10.1002/14651858.CD000180.pub2[published Online First: Epub Date]|.
2. ACOG Committee opinion. Number 267, January 2002: exercise during pregnancy and the postpartum period. Obstet Gynecol 2002;**99**(1):171-3 doi: S0029784401017495 [pii][published Online First: Epub Date]|.
3. Davies GA, Wolfe LA, Mottola MF, MacKinnon C. Joint SOGC/CSEP clinical practice guideline: exercise in pregnancy and the postpartum period. Can J Appl Physiol 2003;**28**(3):330-41
4. (RCOG) RCoOaG. Exercise in pregnancy. Secondary Exercise in pregnancy [HTML] January 2006 2006. http://www.rcog.org.uk/womens-health/clinical-guidance/exercise-pregnancy.
5. Physical activity guidelines for Americans. Okla Nurse 2008;**53**(4):25
6. Haskell WL, Lee IM, Pate RR, et al. Physical activity and public health: updated recommendation for adults from the American College of Sports Medicine and the American Heart Association. Circulation 2007;**116**(9):1081-93 doi: CIRCULATIONAHA.107.185649 [pii]

10.1161/CIRCULATIONAHA.107.185649[published Online First: Epub Date]|.
7. Sallis JF, Patterson TL, Buono MJ, Nader PR. Relation of cardiovascular fitness and physical activity to cardiovascular disease risk factors in children and adults. Am J Epidemiol 1988;**127**(5):933-41
8. Ekelund U, Luan JA, Sherar LB, et al. Moderate to Vigorous Physical Activity and Sedentary Time and Cardiometabolic Risk Factors in Children and Adolescents. Jama-J Am Med Assoc 2012;**307**(7):704-12
9. Jayet PY, Rimoldi SF, Stuber T, et al. Pulmonary and systemic vascular dysfunction in young offspring of mothers with preeclampsia. Circulation 2010;**122**(5):488-94
10. Lawlor DA, Macdonald-Wallis C, Fraser A, et al. Cardiovascular biomarkers and vascular function during childhood in the offspring of mothers with hypertensive disorders of pregnancy: findings from the Avon Longitudinal Study of Parents and Children. Eur Heart J 2011 doi: 10.1093/eurheartj/ehr300[published Online First: Epub Date]|.
11. Lawlor DA, Fraser A, Lindsay RS, et al. Association of existing diabetes, gestational diabetes and glycosuria in pregnancy with macrosomia and offspring body mass index, waist and fat mass in later childhood: findings from a prospective pregnancy cohort. Diabetologia 2010;**53**(1):89-97 doi: 10.1007/s00125-009-1560-z[published Online First: Epub Date]|.
12. Dabelea D, Hanson RL, Lindsay RS, et al. Intrauterine exposure to diabetes conveys risks for type 2 diabetes and obesity: a study of discordant sibships. Diabetes 2000;**49**(12):2208-11
13. Clapp JF, 3rd. Morphometric and neurodevelopmental outcome at age five years of the offspring of women who continued to exercise regularly throughout pregnancy. The Journal of pediatrics 1996;**129**(6):856-63
14. Clapp JF, 3rd, Simonian S, Lopez B, Appleby-Wineberg S, Harcar-Sevcik R. The one-year morphometric and neurodevelopmental outcome of the offspring of women who continued to exercise regularly throughout pregnancy. American journal of obstetrics and gynecology 1998;**178**(3):594-9
15. Pomeroy J, Renstr√∂m F, Gradmark AM, et al. Maternal Physical Activity and Insulin Action in Pregnancy and Their Relationships With Infant Body Composition. Diabetes Care 2012 doi: 10.2337/dc12-0885[published Online First: Epub Date]|.
16. Camilla Schou Andersen MJ, Michael Gamborg, Thorkild I. A. Sørensen, and Ellen Aagaard Nohr. Maternal Recreational Exercise during Pregnancy in relation to Children's BMI at 7 Years of Age. International Journal of Pediatrics 2012;**2012**:8 doi: 10.1155/2012/920583[published Online First: Epub Date]|.

For peer review only - http://bmjopen.bmj.com/site/about/guidelines.xhtml

17. Danielsen I, Granstrom C, Rytter D, et al. Does physical activity during pregnancy adversely influence markers of the metabolic syndrome in adult offspring? A prospective study over two decades. J Epidemiol Community Health 2013;**67**(8):648-54 doi: 10.1136/jech-2012-202017

jech-2012-202017 [pii][published Online First: Epub Date]|.

18. Boyd A, Golding J, Macleod J, et al. Cohort Profile: the 'children of the 90s'--the index offspring of the Avon Longitudinal Study of Parents and Children. International journal of epidemiology 2013;**42**(1):111-27 doi: 10.1093/ije/dys064

dys064 [pii][published Online First: Epub Date]|.

19. Fraser A, Macdonald-Wallis C, Tilling K, et al. Cohort Profile: the Avon Longitudinal Study of Parents and Children: ALSPAC mothers cohort. International journal of epidemiology 2013;**42**(1):97-110 doi: 10.1093/ije/dys066

dys066 [pii][published Online First: Epub Date]|.

20. Ainsworth BE, Haskell WL, SD H, et al. Compendium of Physical Activities: a second update of codes and MET values. Med Sci Sports Exerc 2011

21. Liu J, Blair SN, Teng Y, Ness AR, Lawlor DA, Riddoch C. Physical activity during pregnancy in a prospective cohort of British women: results from the Avon longitudinal study of parents and children. European journal of epidemiology 2011;**26**(3):237-47 doi: 10.1007/s10654-010-9538-1[published Online First: Epub Date]|.

22. Stata Statistical Software: Release 11 [program], 2009.

23. Clinical Guidelines on the Identification, Evaluation, and Treatment of Overweight and Obesity in Adults--The Evidence Report. National Institutes of Health. Obes Res 1998;**6 Suppl 2**:51S-209S

24. Riddoch CJ, Leary SD, Ness AR, et al. Prospective associations between objective measures of physical activity and fat mass in 12-14 year old children: the Avon Longitudinal Study of Parents and Children (ALSPAC). Brit Med J 2009;**339** doi: ARTN b4544

DOI 10.1136/bmj.b4544[published Online First: Epub Date]|.

For peer review only - http://bmjopen.bmj.com/site/about/guidelines.xhtml

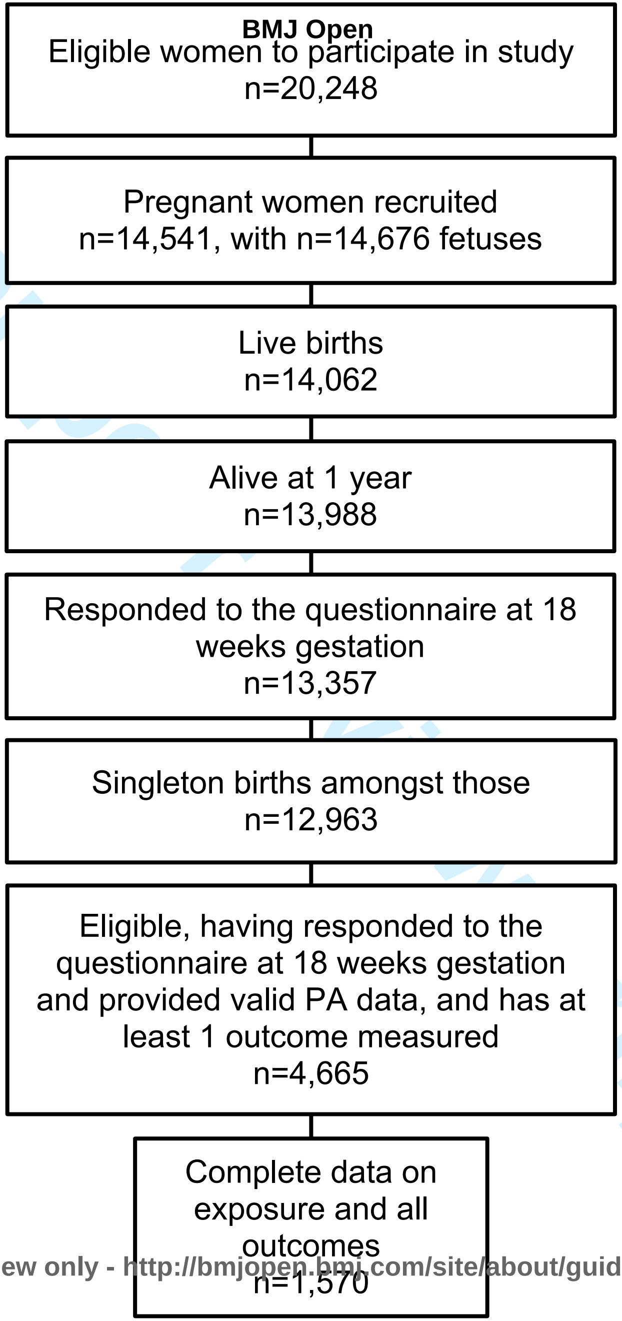

For peer review only - http://bmjopen.bmj.com/site/about/guidelines.xhtml

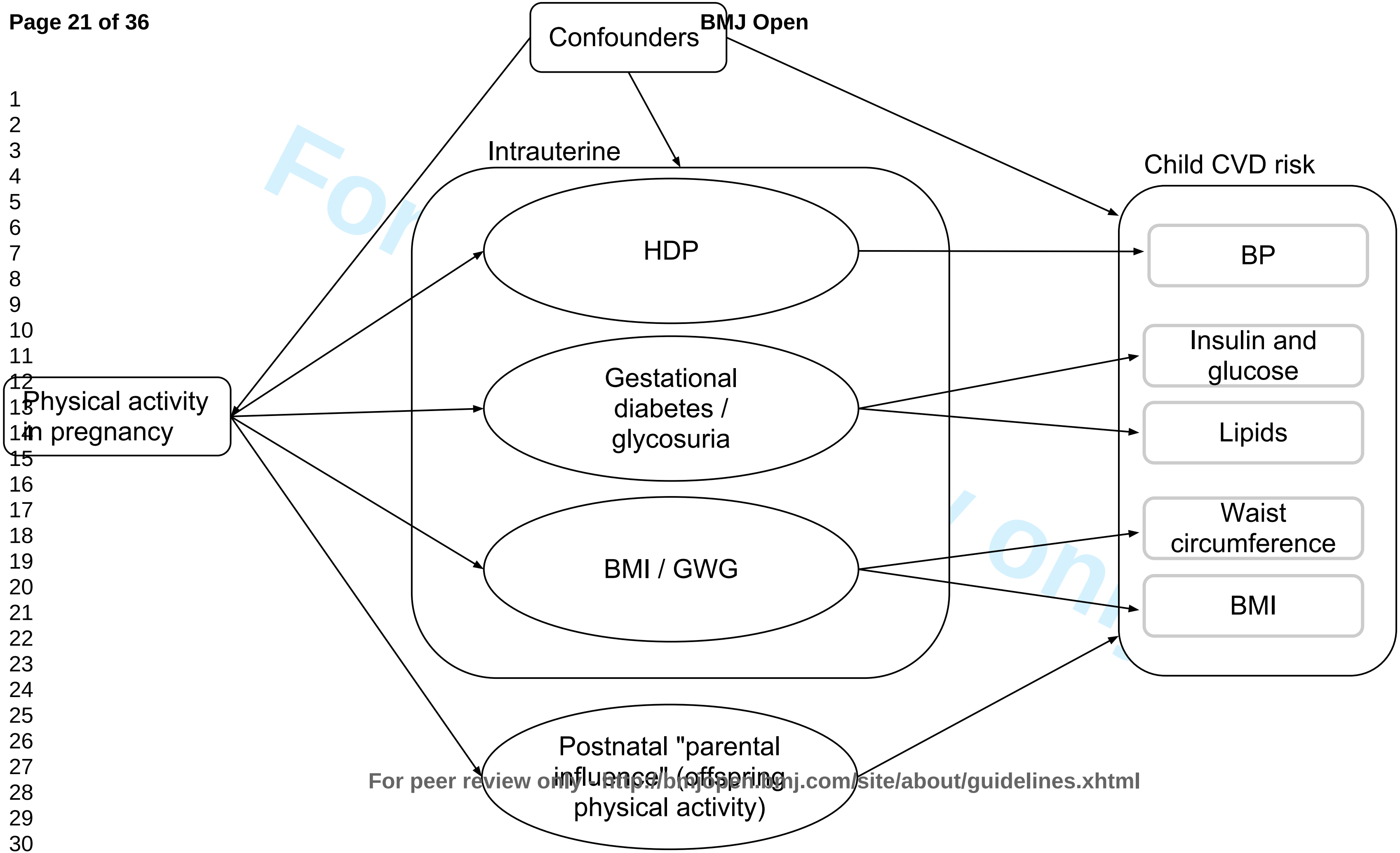
Confounders
Intrauterine
Child CVD risk
HDP
BP
Gestational diabetes / glycosuria
Insulin and glucose
Lipids
Physical activity in pregnancy
BMI / GWG
Waist circumference
BMI
Postnatal "parental influence" (offspring physical activity)

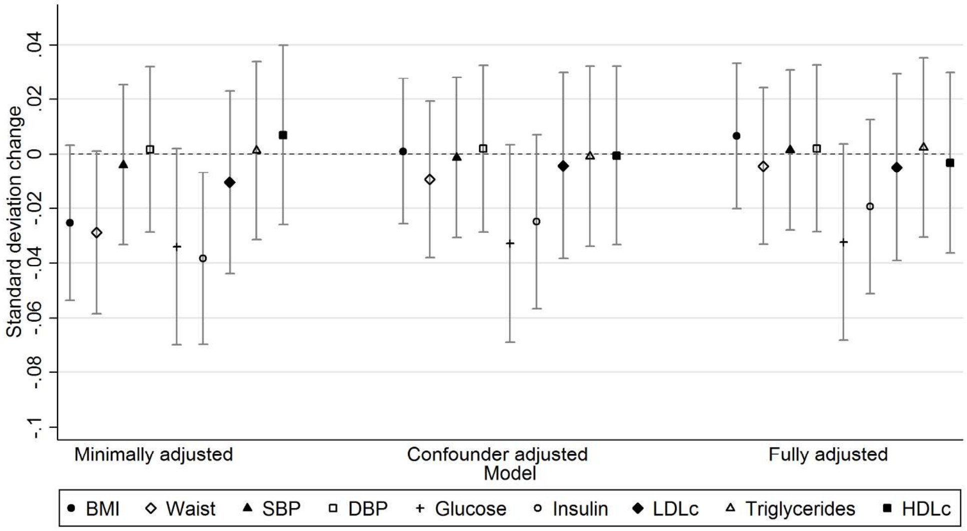

846x476mm (72 x 72 DPI)

review only

Physical activity during pregnancy and offspring cardiovascular risk factors: findings from a prospective cohort study

Supplementary Material

Louise A C Millard, MSc[1,2*], Debbie A Lawlor, PhD [1,2], Abigail Fraser, PhD [1,2], Laura D Howe, PhD[1,2]

1 – MRC Integrative Epidemiology Unit at the University of Bristol,
2 – School of Social and Community Medicine, University of Bristol

*Corresponding author.
Correspondence to: LAC Millard, School of Social and Community Medicine, University of Bristol, Oakfield House, Bristol, UK, BS8 2BN, [louise.millard@bristol.ac.uk], tel +44 117 331 0084, fax: +44 117 331 0123

For peer review only

SUPPLEMENTARY METHODS

Measures used in this study

Cardiovascular risk factors in the offspring Weight and height were measured with the child in light clothing and without shoes. Weight was measured to the nearest 0.1 kg with Tanita scales and height to the nearest 0.1cm with a Harpenden stadiometer. BMI was calculated as weight in kilograms divided by height in metres squared. A flexible tape was used to measure waist circumference to the nearest 1 mm at the mid-point between the lower ribs and the pelvic bone. Blood pressure was measured with a Dinamap 9301 Vital Signs Monitor (Morton Medical, London) with the correct cuff size. Two readings of systolic and diastolic blood pressure were recorded, with the child at rest and their arm supported, and we used the mean of the two measures.

Clinic participants were asked to fast overnight for those attending in the morning or for a minimum of six hours for those attending after lunch. Blood samples were immediately spun and frozen at −80°C. Measurements were assayed three to nine months after samples were taken, with no previous freeze-thaw cycles. Plasma lipid concentrations (total cholesterol, triglycerides, and high density lipoprotein cholesterol (HDLc) were measured by modification of the standard Lipid Research Clinics Protocol by using enzymatic reagents for lipid determination. Low density lipoprotein cholesterol (LDLc) concentrations were determined from these with the Friedwald equation (LDLc=total cholesterol−HDLc+triglycerides×0.45 mmol/l)). Insulin was measured with an enzyme linked immunosorbent assay (ELISA) (Mercodia, Uppsala, Sweden) that does not cross-react with proinsulin, and plasma glucose was measured with an automated assay.

Assessment of potential mediators

Hypertensive disorder of pregnancy (HDP) was assessed using data abstracted from obstetric records that included every measurement of systolic blood pressure (SBP), diastolic blood pressure (DBP), and proteinuria recorded and the corresponding gestational age at the time of these measurements. We applied the International Society for the Study of Hypertension in Pregnancy [1] criteria to all of the clinic data in order to determine women with pre-eclampsia and those with gestational hypertension, and categorized the mothers into one of three mutually exclusive categories; no HDP, gestational hypertension, or pre-eclampsia.

Gestational weight gain was calculated from a linear spline multilevel model developed using all available pregnancy weight measurements (see [2] for further details). We use 3 gestational weight gain variables; 0-18 weeks, 18-28 weeks, 28 weeks onwards, where the 0-18 week variable is used as a confounder (see below) because it refers to the time before the exposure measurement (18 weeks antenatal) whereas the gestational weight gain variables from 18 weeks gestation onwards are used as potential mediators because they refer to the time between exposure and outcome measurement.

At recruitment, women were asked about existing diabetes and any previous history of gestational diabetes. Research midwives abstracted information on clinical diagnoses of gestational diabetes and glycosuria from the antenatal, pregnancy and postnatal medical records using a standard protocol. Using these data, we classified the women into one of four mutually exclusive categories: no evidence of glycosuria or diabetes; existing diabetes before the pregnancy; gestational diabetes (i.e. a diagnosis in the medical records of gestational diabetes in any woman with no history of existing diabetes); and glycosuria (i.e. ++ glycosuria on two occasions in women with no evidence of existing diabetes or gestational diabetes).

Child's physical activity was measured using an accelerometer (MTI Actigraph AM7164 2.2) worn over a period of 7 days. The accelerometer is worn around the waist and detects acceleration and deceleration in a vertical plane as a combined function of movement frequency and intensity. A measure of time spent in moderate and vigorous physical activity (MVPA, equivalent to brisk walking or running) was calculated and averaged over valid days. The accelerometer must have been worn for at least 10 hours a day for at least three days after deletion of missing data to be considered valid [3-5]. To maximize our sample size for this analysis, we used a multilevel model to predict MVPA at exactly 14 years for all participants who had accelerometer measures from at least one of the following time points: age 11, age 13, and age 15. The multilevel model (two levels: occasion and individual) uses all available data from all eligible participants (those with one or more measure) under a missing at random assumption to predict MVPA for all participants, assuming linear rates of change between 11-13 and 13-15 years. Although the model generates values of predicted MVPA between 11 and 15 years old, we included only predicted MVPA at 14 years in our model since there was a high correlation between predicted levels of MVPA at all ages. MLwiN [6] and the runmlwin [7] Stata command were used for the multilevel model.

Gestational age and infant birthweight were recorded in the delivery room and abstracted from obstetric records and/or birth notifications.

For peer review only - http://bmjopen.bmj.com/site/about/guidelines.xhtml

Potential confounders

Maternal weight and height At enrolment (questionnaire at approximately 12 weeks gestation), the mother was asked to record her height and pre-pregnancy weight. Maternal pre pregnancy body mass index (BMI) was calculated by dividing her weight in kilograms by her height in meters squared.

Child age & gender Child age was recorded in months as they arrived for the age 15 year clinic. Infant sex was recorded in the delivery room or abstracted from obstetric records or birth notifications.

Household social class The mother recorded the occupation of both herself and her partner in a questionnaire at 32 weeks gestation, which were used to allocate them to social class groups (I, II, III manual, III non-manual, IV, V) using the 1991 Office of Population, Censuses and Surveys classification; the highest class of the mother and her partner was used in analysis.

Maternal education A questionnaire at 32 weeks gestation asked mothers to report their educational attainment. Maternal educational attainment was categorized as below O-level (ordinary level; exams taken in different subjects usually at age 15–16 at the completion of legally required school attendance, equivalent to today's General Certificate of Secondary Education), O-level only, A-level (advanced-level; exams taken in different subjects usually at age 18), or university degree or above.

Ethnicity A binary variable denoting offspring white or non-white ethnicity was used, where mixed was recoded as non-white due to the small numbers in the mixed category (n=3).

Parity Parity was obtained from obstetric records. Mothers with parity of two or more were grouped into a single category.

Maternal smoking during pregnancy The mother was asked about her smoking habits on two occasions during pregnancy (the 18 week and 32 week antenatal questionnaires), and a dichotomous variable was created for any smoking during pregnancy.

Previous hypertension Mothers were asked about their history of high blood pressure with a yes/no question in a self-administered questionnaire completed at 12 weeks gestation.

Missing data

We used multivariate multiple imputation in Stata as described by Royston [8]. We carried out 20 cycles of regression switching and generated 20 imputation data sets. All variables included in our analyses were used to impute the missing data. The multiple multivariate imputation approach creates a number of copies of the data (in this case, we generated 20 copies) in which missing values are imputed by chained equations [8]. The main analysis results are obtained by averaging across the results from each of these 20 data sets using Rubin's rules, which ensure that the standard errors for any regression coefficients take account of uncertainty in the imputations as well as uncertainty in the estimation [8].

To inform the imputation we included additional variables: cardiovascular disease (CVD) risk factors (BMI and waist circumference at age 11, and SBP, DBP, low density lipoprotein cholesterol (LDLc), high density lipoprotein cholesterol (HDLc), triglycerides at age 9), physical activity at age 12 and measures predictive of missingness of CVD risk factors (paternal education, child total fat mass (age 9), paternal height, maternal age at delivery and birth length). The following variables were log transformed to produce distributions that were approximately normally to improve the imputation: child BMI, waist circumference, LDLc, HDLc and triglycerides age 15, physical activity age 12 and 14, total fat mass and triglycerides age 9, BMI and waist circumference age 11 and maternal BMI.

For peer review only

SUPPLEMENTARY RESULTS

Supplemental Table 1 Physical activity MET scores

Activity	MET score	Number of hours equating to mean MET score [a]
Jogging	7.0	2.3
Brisk walking	3.8	4.2
Swimming	6.0	2.6
Antenatal exercise	2.0	7.9
Keep fit exercise	3.5	4.5
Cycling	5.5	2.9
Aerobics	6.5	2.4
Tennis	4.5	3.5
Yoga	2.5	6.3
Weight training	3.0	5.3
Squash	12.0	1.3
Other	3.5	4.5

[a] Average MET score = 15.8 hours per week

For peer review only

Supplemental Table 2 Summary of variable distributions for observed and imputed data

		Number of participants with non-missing data	Observed data (all individuals) (n (%) or mean (standard deviation (SD)))	Observed data (individuals with complete data, N=1,570) (n (%) or mean (SD))	Imputed data [N= 4,665] (n (%) or mean (standard error)) [c]
Parity (n, %)	0	4633	2259 (48.8)	793 (50.5)	2277 (48.8)
	1		1598 (34.5)	553 (35.2)	1608 (34.5)
	2+		776 (16.8)	224 (14.3)	781 (16.7)
Household	i	4466	761 (17.0)	296 (18.9)	777 (16.7)
social class	ii		2058 (46.1)	766 (48.8)	2123 (45.5)
(n)	iiin		1074 (24.1)	354 (22.5)	1134 (24.3)
	iiim		400 (9.0)	108 (6.9)	438 (9.4)
	iv		173 (3.9)	46 (2.9)	194 (4.2)
Mothers	< o-level	4559	875 (19.2)	241 (15.4)	909 (19.5)
Education	o-level		1595 (35.0)	524 (33.4)	1633 (35.0)
(n)	a-level		1292 (28.3)	486 (31.0)	1315 (28.2)
	degree or above		797 (17.5)	319 (20.3)	808 (17.3)
Child Ethnicity white (n)		4649	4450 (95.7)	1516 (96.6)	4465 (95.7)
Maternal pre-pregnancy weight, kg (mean, SD)		4350	60.56 (11.91)	59.90 (11.18)	60.48 (0.18)
Maternal pre-pregnancy height, cm (mean)		4497	164.33 (6.68)	164.44 (6.72)	164.30 (0.10)
Maternal gestational weight gain 0 – 18 weeks, kg/week (mean)		4350	0.31 (0.17)	0.32 (0.17)	0.31 (0.00)
Mother previous hypertension, yes (n)		4087	190 (4.7)	60 (3.8)	216 (4.6)
Mother smokes in pregnancy, yes (n)		4653	762 (16.4)	238 (15.2)	764 (16.4)
Child gender, female (n)		4665	2436 (52.2)	801 (51.0)	2436 (52.2)
HDP (n)	None	4644	3885 (83.7)	1352 (86.1)	3903 (83.7)
	Pre-eclampsia		89 (1.9)	20 (1.3)	90 (1.9)
	Gestational hypertension		670 (14.4)	198 (12.6)	673 (14.4)
Maternal	None	4548	4358 (95.8)	1515 (96.5)	4468 (95.8)
diabetes	Existing diabetes		20 (0.4)	5 (0.3)	22 (0.5)
(n)	Gestational diabetes		27 (0.6)	8 (0.5)	28 (0.6)
	Glycosuria		143 (3.1)	42 (2.7)	147 (3.2)
Maternal physical activity, MET hours/week (mean) [a]		4665	16.41 (15.32)	17.02 (15.87)	16.41 (0.22)
Child BMI age 15, kg/m^2 (mean)		4603	21.44 (3.53)	21.19 (3.13)	21.45 (0.05)

For peer review only

Child waist circumference age 15, cm (mean)	3828	76.67 (8.92)	76.54 (8.58)	77.15 (0.14)
Child SBP age 15, mmHg (mean)	4345	123.21 (10.84)	123.52 (10.88)	123.28 (0.17)
Child DBP age 15, mmHg (mean)	4345	67.63 (8.74)	66.63 (8.45)	67.64 (0.13)
Child glucose age 15, mmol/L (mean)	2957	5.21 (0.39)	5.20 (0.37)	5.21 (0.01)
Child insulin age 15, IU/L (mean)	2953	10.32 (6.50)	9.60 (4.74)	10.34 (0.11)
Child LDLc age 15, mmol/L (mean)	2957	2.09 (0.56)	2.09 (0.54)	2.09 (0.01)
Child triglycerides age 15, mmol/L (mean)	2957	0.84 (0.39)	0.81 (0.33)	0.84 (0.01)
Child HDLc age 15, mmol/L (mean)	2957	1.28 (0.29)	1.30 (0.29)	1.29 (0.01)
Child age at 15 year clinic, weeks (mean)	4665	808.22 (17.45)	805.00 (12.49)	808.22 (0.26)
Child physical activity age 14 (mean) [b]	3938	1.45 (0.62)	1.48 (0.63)	1.45 (0.01)
Maternal gestational weight gain 18 - 28/40, kg/week (mean)	4350	0.54 (0.17)	0.53 (0.16)	0.53 (0.00)
Maternal gestational weight gain 28/40 onwards kg/week (mean)	4350	0.47 (0.19)	0.46 (0.19)	0.47 (0.00)
Birthweight, grams (mean)	4603	3440.06 (523.94)	3497.28 (472.43)	3439.64 (7.69)
Gestational age, weeks (mean)	4494	32.50 (1.21)	32.51 (1.12)	32.50 (0.02)

[a] Metabolic equivalent maternal physical activity
[b] Mean daily minutes of MVPA (predicted)
[c] Multivariate multiply imputed data over 20 imputation data sets of all mother-offspring pairs with a value of physical activity during pregnancy (exposure) and at least one offspring cardiovascular risk factor age 15.5 (outcomes)

For peer review only - http://bmjopen.bmj.com/site/about/guidelines.xhtml

For peer review only

Supplemental Table 3 Association of maternal physical activity with potential mediators of the relationship between physical activity during pregnancy and offspring cardiovascular risk factors: using multivariate multiply imputed data (N = 4,665) of individuals with data for maternal physical activity (exposure) and at least one outcome

Potential mediator	Mediator category	Change per 1 SD greater maternal metabolic equivalent physical activity during pregnancy
**Odds ratio per SD greater maternal physical activity during pregnancy, null value = 1**		
Hypertensive Disorders of Pregnancy	None	Reference
	Pre-eclampsia	-0.04 (-0.25 to 0.17)
	Gestational hypertension	-0.04 (-0.12 to 0.05)
Diabetes	None	Reference
	Existing diabetes	0.18 (-0.23 to 0.58)
	Gestational diabetes	-0.29 (-0.73 to 0.15)
	Glycosuria	-0.03 (-0.20 to 0.14)
**Mean difference per SD greater maternal physical activity during pregnancy, null value = 0**		
Gestational weight gain (SD)	18 - 28 weeks	-0.02 (-0.05 to 0.01)
	28 weeks onwards	-0.01 (-0.04 to 0.02)
Gestational age (SD)		-0.02 (-0.05 to 0.01)
Birthweight (SD)		-0.01 (-0.04 to 0.02)
Offspring moderate to vigorous physical activity age 14 (SD)		0.06 (0.03 to 0.09)

SD: Standard deviation

For peer review only

Supplemental Table 4 Association between maternal physical activity during pregnancy and offspring cardiovascular risk factors: using multivariate multiply imputed data (N = 4,665) of individuals with data for maternal physical activity (exposure) and at least one outcome

	Change per 1 standard deviation (SD) greater maternal metabolic equivalent physical activity (95% confidence intervals)					
Outcome	Minimal adjustment for child age and gender	Adjusted for confounders: household social class, maternal education, ethnicity, parity, maternal smoking during pregnancy, previous hypertension, maternal pre-pregnancy BMI and gestational weight gain 0-18 weeks	Adjusted for confounders and gestational diabetes, HDP, gestational weight gain	Adjusted for confounders, gestational diabetes, HDP, gestational weight gain, gestational age, and birthweight	Adjusted for confounders and offspring physical activity	Adjusted for all confounders and mediators
**Mean difference (in units specified in first column) per 1 SD greater maternal physical activity during pregnancy**						
BMI, kg/m^2	-0.089 (-0.190 to 0.011)	0.004 (-0.091 to 0.098)	0.004 (-0.090 to 0.099)	0.006 (-0.088 to 0.100)	0.021 (-0.073 to 0.115)	0.023 (-0.071 to 0.117)
Waist circumference, cm	-0.271 (-0.552 to 0.011)	-0.088 (-0.358 to 0.181)	-0.085 (-0.354 to 0.184)	-0.082 (-0.351 to 0.186)	-0.048 (-0.317 to 0.221)	-0.042 (-0.311 to 0.227)
SBP, mmHg	-0.044 (-0.360 to 0.272)	-0.014 (-0.332 to 0.303)	-0.012 (-0.329 to 0.305)	-0.008 (-0.325 to 0.309)	0.012 (-0.307 to 0.330)	0.017 (-0.301 to 0.334)
DBP, mmHg	0.015 (-0.251 to 0.280)	0.017 (-0.251 to 0.284)	0.019 (-0.248 to 0.287)	0.022 (-0.246 to 0.290)	0.014 (-0.254 to 0.282)	0.018 (-0.250 to 0.286)
Glucose, mmol/L	-0.013 (-0.027 to 0.001)	-0.013 (-0.027 to 0.001)	-0.013 (-0.026 to 0.001)	-0.013 (-0.027 to 0.001)	-0.013 (-0.026 to 0.001)	-0.012 (-0.026 to 0.001)
LDLc, mmol/L	-0.006 (-0.025 to 0.013)	-0.002 (-0.022 to 0.017)	-0.003 (-0.022 to 0.017)	-0.003 (-0.022 to 0.017)	-0.002 (-0.022 to 0.017)	-0.003 (-0.022 to 0.017)
HDLc, mmol/L	0.002 (-0.008 to 0.012)	0.000 (-0.010 to 0.010)	0.000 (-0.010 to 0.010)	0.000 (-0.010 to 0.010)	-0.001 (-0.011 to 0.009)	-0.001 (-0.011 to 0.009)
**Percentage difference per 1 SD greater maternal physical activity during pregnancy**						
Insulin, % difference	-1.90 (-3.50 to -0.30)	-1.20 (-2.80 to 0.40)	-1.20 (-2.80 to 0.40)	-1.20 (-2.80 to 0.40)	-1.00 (-2.60 to 0.60)	-1.00 (-2.60 to 0.60)
Triglycerides, %	0.00 (-1.20 to 1.30)	0.00 (-1.30 to 1.20)	0.00 (-1.30 to 1.30)	0.00 (-1.30 to 1.30)	0.00 (-1.20 to 1.30)	0.00 (-1.20 to 1.40)

For peer review only - http://bmjopen.bmj.com/site/about/guidelines.xhtml

difference						

For peer review only - http://bmjopen.bmj.com/site/about/guidelines.xhtml

Supplemental Fig. 1 BMI interaction analysis showing associations of maternal physical activity with child offspring CVD outcomes for overweight and not overweight individuals using multiply imputed sample (N=4,665)

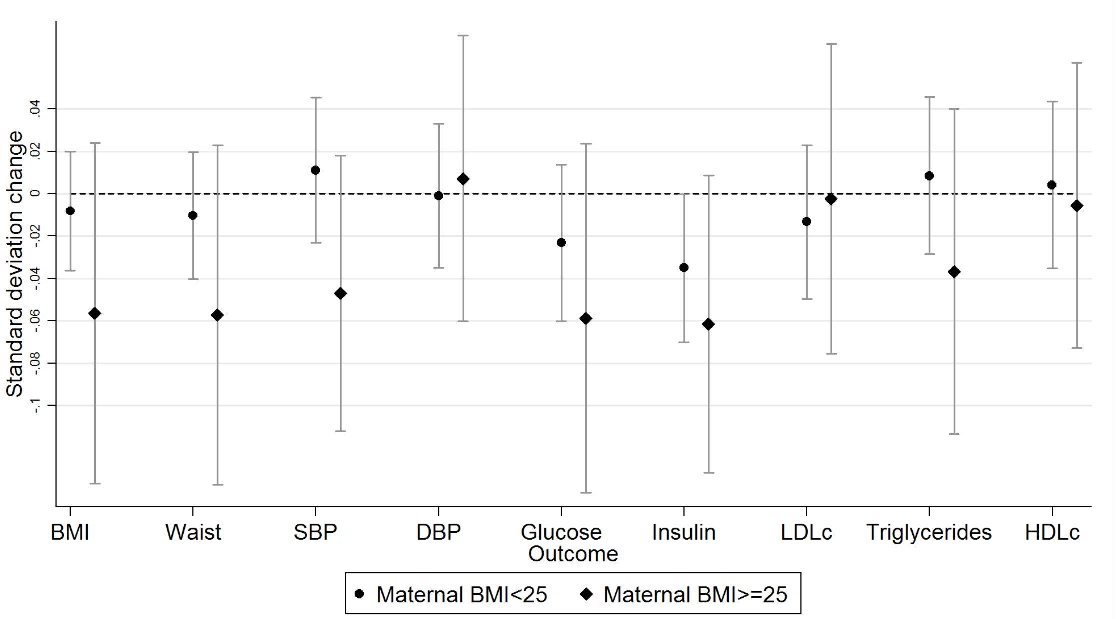

Mean standard deviation difference (and 95% confidence intervals) in offspring cardiovascular risk factors (or logged distribution for insulin and triglycerides) for a 1 standard deviation increase in metabolic equivalent maternal physical activity during pregnancy

For peer review only - http://bmjopen.bmj.com/site/about/guidelines.xhtml

Supplemental Fig. 2 Associations of maternal metabolic equivalent maternal physical activity during pregnancy with child BMI using complete case and multiply imputed samples

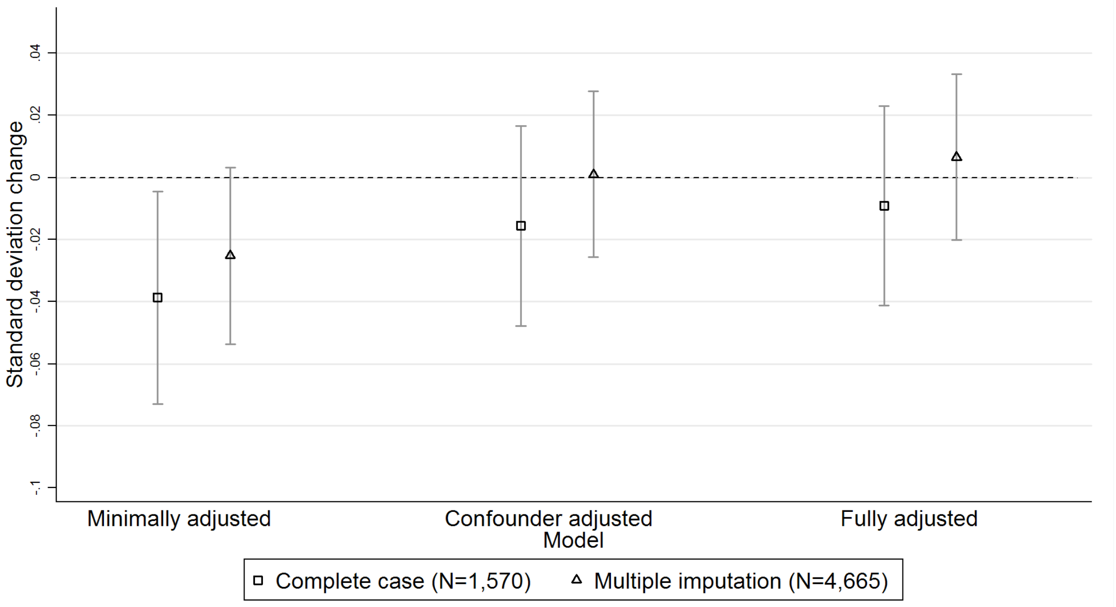

Mean standard deviation difference (and 95% confidence intervals) in offspring BMI per 1 standard deviation increase in metabolic equivalent maternal physical activity during pregnancy

Multiple imputation: 20 imputations (Stata ice command)
Multiple imputation (N=4,665): all mother-offspring pairs with a value of physical activity during pregnancy and at least one offspring cardiovascular risk factor age 15.5
Complete case: Original data excluding individuals with any missing data for all variables per outcome

For peer review only - http://bmjopen.bmj.com/site/about/guidelines.xhtml

Supplemental Fig. 3 Associations of maternal metabolic equivalent maternal physical activity during pregnancy with child blood insulin levels using complete case and multiply imputed samples

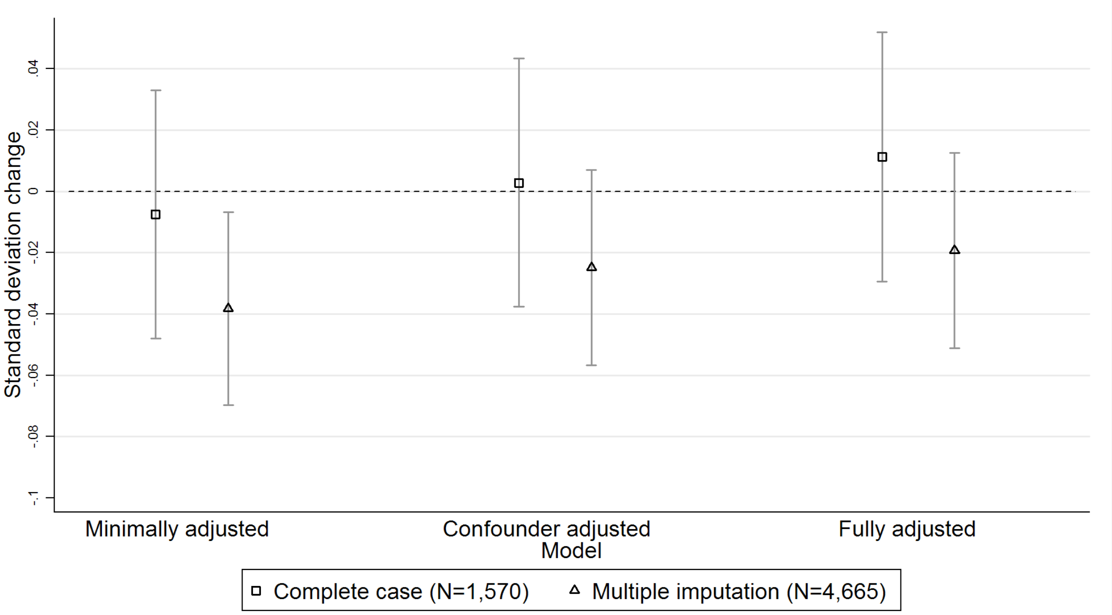

Mean standard deviation difference (and 95% confidence intervals) in log transformed offspring insulin per 1 standard deviation increase in metabolic equivalent maternal physical activity during pregnancy

Multiple imputation: 20 imputations (Stata ice command)
Multiple imputation (N=4,665): all mother-offspring pairs with a value of physical activity during pregnancy and at least one offspring cardiovascular risk factor age 15.5
Complete case: Original data excluding individuals with any missing data for all variables per outcome

For peer review only - http://bmjopen.bmj.com/site/about/guidelines.xhtml

Supplemental Fig. 4 Associations of maternal metabolic equivalent maternal physical activity during pregnancy with child blood glucose levels using complete case and multiply imputed samples

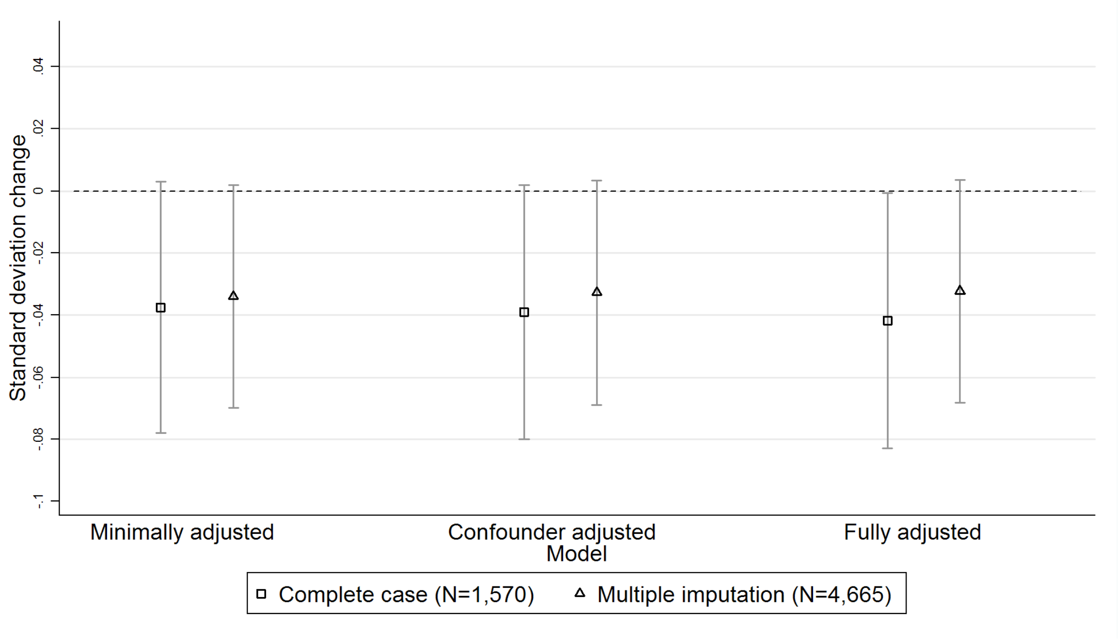

Mean standard deviation difference (and 95% confidence intervals) in offspring glucose per 1 standard deviation increase in metabolic equivalent maternal physical activity during pregnancy

Multiple imputation: 20 imputations (Stata ice command)
Multiple imputation (N=4,665): all mother-offspring pairs with a value of physical activity during pregnancy and at least one offspring cardiovascular risk factor age 15.5
Complete case: Original data excluding individuals with any missing data for all variables per outcome

For peer review only - http://bmjopen.bmj.com/site/about/guidelines.xhtml

REFERENCES

1. Davey DA, MacGillivray I. The classification and definition of the hypertensive disorders of pregnancy. American journal of obstetrics and gynecology 1988;**158**(4):892-8 doi: 0002-9378(88)90090-7 [pii][published Online First: Epub Date]|.
2. Fraser A, Tilling K, Macdonald-Wallis C, et al. Association of maternal weight gain in pregnancy with offspring obesity and metabolic and vascular traits in childhood. Circulation 2010;**121**(23):2557-64 doi: 10.1161/CIRCULATIONAHA.109.906081[published Online First: Epub Date]|.
3. Mattocks C, Leary S, Ness A, et al. Calibration of an accelerometer during free-living activities in children. Int J Pediatr Obes 2007;**2**(4):218-26 doi: 781692295 [pii]

10.1080/17477160701408809[published Online First: Epub Date]|.
4. Mattocks C, Ness A, Leary S, et al. Use of Accelerometers in a Large Field-Based Study of Children: Protocols, Design Issues, and Effects on Precision. J Phys Act Health 2008;**5**:S98-S111
5. Riddoch CJ, Mattocks C, Deere K, et al. Objective measurement of levels and patterns of physical activity. Arch Dis Child 2007;**92**(11):963-69 doi: Doi 10.1136/Adc.2006.112136[published Online First: Epub Date]|.
6. MLwiN Version 2.1, Centre for Multilevel Modelling, University of Bristol. [program]. Bristol, 2009.
7. runmlwin: Stata module for fitting multilevel models in the MLwiN software package. Centre for Multilevel Modelling, University of Bristol. [program]. Bristol, 2009.
8. Royston P. Multiple imputation of missing values. Stata J, 2004:227–41.

For peer review only

Physical activity during pregnancy and offspring cardiovascular risk factors: findings from a prospective cohort study

Journal:	*BMJ Open*
Manuscript ID:	bmjopen-2013-003574.R1
Article Type:	Research
Date Submitted by the Author:	22-Aug-2013
Complete List of Authors:	Millard, Louise; University of Bristol, School of Social and Community Medicine<br>Lawlor, Debbie; Department of Social Medicine, University of Bristol, Department of Social Medicine<br>Fraser, Abigail; University of Bristol, School of Social and Community Medicine<br>Howe, Laura; University of Bristol, Social Medicine
**Primary Subject Heading**:	Epidemiology
Secondary Subject Heading:	Paediatrics, Cardiovascular medicine, Obstetrics and gynaecology
Keywords:	Paediatric cardiology < PAEDIATRICS, PAEDIATRICS, EPIDEMIOLOGY

SCHOLARONE™
Manuscripts

For peer review only

Physical activity during pregnancy and offspring cardiovascular risk factors: findings from a prospective cohort study

Louise A C Millard, MSc[1,2*], Debbie A Lawlor, PhD [1,2], Abigail Fraser, PhD [1,2], Laura D Howe, PhD[1,2]

1 – MRC Integrative Epidemiology Unit at the University of Bristol,
2 – School of Social and Community Medicine, University of Bristol

*Corresponding author.
Correspondence to: LAC Millard, School of Social and Community Medicine, University of Bristol, Oakfield House, Bristol, UK, BS8 2BN, [louise.millard@bristol.ac.uk], tel +44 117 331 0084, fax: +44 117 331 0123

For peer review only

For peer review only - http://bmjopen.bmj.com/site/about/guidelines.xhtml

ABSTRACT

Objectives

The long term consequences of maternal physical activity during pregnancy for offspring cardiovascular health are unknown. We examined the association of maternal self-reported physical activity in pregnancy (18 weeks gestation) with offspring cardiovascular risk factors at age 15.

Design

Prospective cohort study.

Setting

The Avon Longitudinal Study of Parents and Children (ALSPAC).

Participants

4,665 maternal-offspring pairs (based on a sample with multivariate multiple imputation to deal with missing data) from the Avon Longitudinal Study of Parents and Children, a prospective cohort based in the South West of England with mothers recruited in pregnancy in 1991-1992.

Primary and secondary outcome measures

Offspring cardiovascular risk factors at age 15; body mass index, waist circumference, systolic blood pressure, diastolic blood pressure, glucose, insulin, low density lipoprotein cholesterol, high density lipoprotein cholesterol and triglycerides.

Results

Greater maternal physical activity was associated with lower BMI, waist circumference, glucose and insulin in unadjusted analyses. The magnitude of associations was generally small with wide confidence intervals, and most associations attenuated towards the null after adjusting for confounders. The strongest evidence of association after adjustment for confounders was for glucose, although the 95% confidence interval for this association includes the null; a one standard deviation greater physical activity during pregnancy was associated with a -0.013 mmol/l difference in offspring glucose levels (equivalent to approximately one third of a standard deviation; 95% confidence interval -0.027 to 0.001 mmol/l).

For peer review only - http://bmjopen.bmj.com/site/about/guidelines.xhtml

Conclusions

Our results suggest that maternal physical activity in pregnancy, measured at 18 weeks gestation, is unlikely to be an important determinant of later offspring cardiovascular health. There was some suggestion of association with offspring glucose, but given that all other associations (including insulin) were null after adjustment for confounders, this result should be interpreted with caution.

For peer review only

For peer review only - http://bmjopen.bmj.com/site/about/guidelines.xhtml

ARTICLE SUMMARY

Article focus

- To examine the association of maternal self-reported physical activity in pregnancy (18 weeks gestation) with offspring cardiovascular risk factors at age 15.

Key messages

- Self-reported physical activity in pregnancy, at 18 weeks gestation, is unlikely to be an important determinant of later offspring cardiovascular health.

Strengths and limitations of this study

- The main strengths of our study are the large sample size, prospective nature of data collection, and the detailed measurement of a wide range of offspring cardiovascular risk factors in adolescence.
- A limitation of our work is that the data on maternal physical activity during pregnancy were self-reported, and thus measurement error is possible. We cannot assess women's physical activity levels before pregnancy, or the extent to which women changed their physical activity levels between the 18 week questionnaire and the end of the pregnancy. Thus it is possible that physical activity later in pregnancy may be a more important exposure for offspring cardiovascular risk.

For peer review only

For peer review only - http://bmjopen.bmj.com/site/about/guidelines.xhtml

INTRODUCTION

There is increasing interest in the potential effects of physical activity during pregnancy on both mother and offspring [1]. Current policy encourages physical activity during pregnancy, with UK guidelines recommending at least 30 minutes of moderate intensity activity at least 4 times a week [2-4], and US guidelines recommending 2 ½ hours each week [5]. However, the evidence regarding potential risks and benefits of physical activity during pregnancy is unclear, with a Cochrane systematic review of randomised controlled trials concluding that regular aerobic exercise during pregnancy (mostly swimming, static cycling, floor exercises) improves women's physical fitness, but that there was insufficient evidence to draw firm conclusions about any other short- or long-term risks or benefits to the woman or her offspring [1].

Physical activity is associated with lower cardiovascular risk in both adults and children [6-8]. It is possible that greater levels of physical activity during pregnancy might improve later offspring cardiovascular health, either through direct intrauterine mechanisms influencing musculoskeletal or other system development or epigenetic changes, or because mothers who are more active in pregnancy are also likely to be so postnatally and might encourage their children to be more active [9-12]. There is some evidence that greater levels of physical activity during pregnancy is associated with later offspring growth and lower adiposity [13-16]. All but one of these studies [16] had a very small sample size, and none were able to assess cardiovascular risk factors other than adiposity in offspring. Furthermore, a recent study [17] found evidence of an adverse effect of physical activity measured at 30 weeks gestation on high density lipoprotein cholesterol, diastolic blood pressure and body mass index (BMI). Thus, it is unclear whether variation in levels of physical activity during pregnancy is (either beneficially or detrimentally) related to long-term cardiovascular health in offspring.

The aim of this study is to investigate the association of physical activity during pregnancy with a range of offspring cardiovascular risk factors (BMI, waist circumference, glucose, insulin, lipids, and blood pressure). We further aim to explore whether any observed associations are explained by measured confounders, or if not, whether they are mediated by intrauterine characteristics such as hypertensive disorders of pregnancy (HDP), gestational diabetes or gestational weight gain, or whether they are mediated by the offspring's own later physical activity levels.

For peer review only - http://bmjopen.bmj.com/site/about/guidelines.xhtml

METHODS

The Avon Longitudinal Study of Parents and Children (ALSPAC) is a prospective, population-based study investigating environmental and other factors that affect the health and development of children. The study methods are described in detail elsewhere [18 19] and on the study website (**http://www.alspac.bris.ac.uk**). In brief, all pregnant women living in the three health districts centered around Bristol, in the South West of England, who had an expected delivery date between April 1, 1991 and December 31, 1992 were invited to take part in the study. Ethics approval for the study was obtained from the ALSPAC Law and Ethics Committee and the Local Research Ethics Committee.

From the 14,062 live births in the study, we restricted analyses to singleton pregnancies of offspring alive at 1 year, and excluded 1,234 women who did not provide data on physical activity during pregnancy, 1 woman with an implausible value of physical activity during pregnancy and 7,063 offspring without data for at least one outcome, yielding a sample size of 4,665 mother-offspring pairs (see Fig. 1~~Fig. 1~~ for the study participant flow diagram).

Physical activity during pregnancy

At 18 weeks gestation women were asked, in a validated questionnaire [20], to report the hours per week that they currently spend doing each of 11 types of leisure time physical activities (brisk walking, swimming, antenatal exercise, keep fit exercise, cycling, aerobics, tennis, yoga, jogging, weight training, squash, other exercise). Based on the responses to these questions, we estimated a weighted activity index (i.e. the weekly total energy expenditure from these activities) by multiplying the published average metabolic equivalent (MET) scores for the reported activities [21] with the estimated hours per week (7 for ‘‘≥7 h’’, 4 for ‘‘2–6 h’’, 0.5 for ‘‘<1 h’’, and 0 for ‘‘never’’) – see Supplemental Table 1 (Online Resource) for example MET scores. MET scores are defined as the ratio of the work metabolic rate to a standard resting metabolic rate of 1.0 $kcal \cdot kg^{-1} \cdot hr^{-1}$ [22]. MET scores were derived from published sources that measured the oxygen cost of each activity, or estimated from the MET scores of similar activities where no studies could be found for this activity [22]. Published MET values are not available for ‘antenatal exercise’ and ‘keep-fit exercise’, thus we assigned 2.0 and 3.5 METs, respectively. Physical activity equating to a score greater than 70 was reported by 46 individuals, which is equivalent to 7 hours per week of jogging; these values were re-coded to 70 in order to prevent them from having undue influence on the results [22].

For peer review only - http://bmjopen.bmj.com/site/about/guidelines.xhtml

Cardiovascular risk factors in the offspring

Offspring cardiovascular risk factors (body mass index (BMI), waist circumference, glucose, insulin, lipids (high density lipoprotein cholesterol (HDLc) and low density lipoprotein cholesterol (LDLc)), and blood pressure) were measured at a research clinic attended at mean age 15.5 years. Weight and height were measured with the child in light clothing without shoes. BMI was calculated as weight in kilograms divided by height in metres squared. A flexible tape was used to measure waist circumference to the nearest 1 mm at the mid-point between the lower ribs and the pelvic bone. Blood pressure was measured with a Dinamap 9301 Vital Signs Monitor (Morton Medical, London) with the correct cuff size. Two readings of systolic and diastolic blood pressure were recorded, with the child at rest and their arm supported, and we used the mean of the two measures. A fasting blood sample was taken and used to measure total cholesterol, triglycerides and HDLc. LDLc concentrations were determined from these with the Friedwald equation (LDLc=total cholesterol−HDLc+triglycerides×0.45 mmol/l)). Insulin was measured with an enzyme linked immunosorbent assay (ELISA) (Mercodia, Uppsala, Sweden) that does not cross-react with proinsulin, and plasma glucose was measured with an automated assay. Further details are provided in Online Resource.

Other measurements

We considered the following to be potential confounding factors: household social class, maternal education, ethnicity, parity (the number of previous pregnancies resulting in a live birth or stillbirth), smoking during pregnancy, previous history of hypertension outside of pregnancy, pre-pregnancy BMI, and gestational weight gain in the first 18 weeks of pregnancy. We also adjusted for the offspring's gender and age at assessment of outcomes in all analyses.

We considered the following to be potential mediating factors: HDP (mothers with pre-eclampsia or gestational hypertension), gestational weight gain (between 18-28 weeks and 28 weeks onwards; see Online Resource for explanation of why these two time points were used), gestational diabetes, offspring gestational age, birthweight and offspring accelerometer-assessed moderate to vigorous physical activity at 14 years. Gestational age and birthweight are plausible mediators of the intrauterine pathways due to their association with both gestational diabetes and HDP, and offspring outcomes [10 11]. Details of the assessment of all of these potential confounders and mediators are provided in Online Resource.

For peer review only - http://bmjopen.bmj.com/site/about/guidelines.xhtml

Statistical Analysis

All analysis was performed with Stata v11.2 [23]. Insulin and triglycerides were positively skewed and so were log transformed. Multivariable linear regression was used to examine the association between the weighted score of maternal physical activity during pregnancy and offspring cardiovascular risk factors. The weighted score of maternal physical activity in pregnancy was standardized to have a mean of zero and variance of one. Coefficients from regression models therefore represent the mean difference in cardiovascular risk factors (in their measured units or a percentage difference for the log transformed insulin and triglyceride outcomes) for a one standard deviation increase in MET physical activity, which is equivalent to approximately 15 MET hours per week, i.e. just over two hours of jogging, or just under four hours of brisk walking. We also repeated all analyses with each cardiovascular outcome scaled per standard deviation of their distribution (or logged distribution for insulin and triglycerides) and present these results in figures. This allowed us to compare the magnitudes of any associations across outcomes. We used tests of interaction to assess whether the associations between maternal physical activity during pregnancy and offspring cardiovascular risk factors differed by child gender or by maternal pre-pregnancy BMI (categorized as underweight/normal weight [BMI less than 25] or overweight/obese [BMI 25 or above]) [24].

Fig. 2~~Fig. 2~~ illustrates the hypothesised pathways between maternal physical activity during pregnancy and offspring cardiovascular risk factors. We carried out several analyses to explore these pathways. We initially investigated minimally adjusted associations, adjusting only for the child's gender and exact age at outcome measurement. Confounder-adjusted models adjusted for child gender, exact age at outcome measurement, household social class, maternal education, ethnicity, parity, smoking during pregnancy, previous hypertension, pre-pregnancy weight and gestational weight gain between 0-18 weeks gestation. The possible mediating mechanisms involved were investigated in two further models: a) all confounders plus intrauterine characteristics – HDP, gestational diabetes and gestational weight gain after 18 weeks, gestational age and birthweight, and b) all confounders plus offspring physical activity (measured objectively using an accelerometer) at age 14. A final model included all potential confounding and mediating factors.

<u>Missing data</u>

We used multivariate multiple imputation (details in Online Resource) to impute missing data for all mother-offspring pairs where the data on maternal physical activity during pregnancy (the exposure) and at least one cardiovascular risk factor at age 15 was observed (the eligible sample, N=4,665). Analyses conducted using this

For peer review only

imputation dataset were considered our main analyses. However, in order to assess the impact of missing data on our results, we compared the results of the main analysis (using imputed datasets) with results from a complete case approach in which we included only individuals with complete data for all outcomes, all confounders and all mediators. Characteristics of participants and levels of missing data for each variable are shown in Supplementary Table 2 (Online Resource).

For peer review only

For peer review only - http://bmjopen.bmj.com/site/about/guidelines.xhtml

RESULTS

There was large inter-individual variation in reported MET-hours per week at 18 weeks gestation. Approximately one third of mothers reported less than 6 MET hours per week, which equates to one hour of swimming, whilst approximately one quarter of women reported over 24 MET hours per week. On average, higher social class, greater maternal education, lower parity, no maternal smoking in pregnancy and lower maternal pre-pregnancy BMI were associated with greater reported physical activity at 18 weeks gestation (Table 1). There was limited evidence that maternal physical activity was associated with most of our a priori specified mediators of the association between physical activity in pregnancy and offspring cardiovascular risk factors; no strong evidence of association was seen for hypertensive disorders of pregnancy, diabetes, gestational weight gain, gestational age, or birth weight, but greater maternal physical activity during pregnancy was associated with greater offspring physical activity at age 14. For each standard deviation increase in reported physical activity during pregnancy, there was a 0.06 SD increase in physical activity in the offspring (95% CI 0.03 to 0.09) (Supplementary Table 3 in Online Resource).

Association between physical activity during pregnancy and offspring cardiovascular risk factors

There was no evidence that the associations between maternal physical activity during pregnancy and offspring cardiovascular risk factors differed either by offspring gender (all *P* for interaction > 0.2) or by maternal pre-pregnancy BMI status (all *P* for interaction > 0.1 see Supplemental Figure 1 in Online Resource). Fig. 3~~Fig. 3~~ and Table 2 show the minimally adjusted, confounder adjusted and mediator (intrauterine characteristics and offspring physical activity) adjusted associations of maternal physical activity during pregnancy with offspring cardiovascular risk factors. More detailed results from models examining each potential mediator separately are given in Supplemental Table 4 in Online Resource).

There was little evidence that maternal physical activity during pregnancy was associated with the offspring cardiovascular risk factors. In minimally adjusted analyses (adjusting only for child gender and age at outcome assessment), higher levels of physical activity were associated with lower offspring BMI, smaller waist circumference, lower glucose, and lower insulin. For all outcomes except insulin, glucose, BMI and waist circumference, the magnitude of coefficients was small and confidence intervals were wide. A one standard deviation increase in physical activity during pregnancy is associated with a lower insulin level in the offspring of -1.90% (95% CI -3.50 to -0.30) and lower glucose level by -0.013 mmol/l (95% CI -0.027 to 0.001).

For peer review only - http://bmjopen.bmj.com/site/about/guidelines.xhtml

Adjustment for measured confounders moved most of the associations towards the null. After adjusting for confounding, one standard deviation increase in physical activity during pregnancy is associated with lower offspring insulin by -1.20% (95% CI -2.80 to 0.40). The borderline association with glucose did not alter after adjusting for confounders. Adjusting for potential mediators had little effect on the estimated associations for any outcome.

Sensitivity of results to missing data

The results based on multivariate multiple imputation were very similar to those based on individuals with complete data (Supplemental Figure 2 for BMI, Supplemental Figure 3 for insulin and Supplemental Figure 4 for glucose are shown as illustrative examples, in Online Resource).

For peer review only

For peer review only - http://bmjopen.bmj.com/site/about/guidelines.xhtml

DISCUSSION

Despite greater maternal physical activity in pregnancy being associated with lower pre-pregnancy BMI and greater offspring physical activity at mean age 14 years, there was no strong evidence that it was related to later offspring cardiovascular risk factors (BMI, waist circumference, blood pressure (systolic blood pressure, diastolic blood pressure), lipids (triglycerides, low density lipoprotein cholesterol, high density lipoprotein cholesterol), and fasting glucose or insulin) at age 15 years using data from a contemporary general population pregnancy cohort. The associations we observed were small, and were generally attenuated with adjustment for measured confounders. The strongest evidence of association was for glucose (one standard deviation greater physical activity in pregnancy was associated with a lower glucose by -0.013 mmol/l (95% confidence interval -0.027 to 0.001), equivalent to approximately one third of a standard deviation), but given that we investigated several outcomes, that the association with all other outcomes including insulin attenuated towards the null after adjustment for confounding, and that no associations were seen for BMI, waist circumference or any other cardiovascular risk factor, this finding should be interpreted with caution and requires replication in other studies.

To our knowledge only one study to date has investigated the association of maternal physical activity during pregnancy with a range of offspring cardiovascular risk factors [17]. This study included 965 Danish participants at age 20, and examined the associations of self-reported physical activity at work, leisure time physical activity, daily amount of walking or cycling, and sport participation between 18 and 30 weeks gestation (measured at 30 weeks gestation), with offspring cardiovascular risk factors. The authors found that higher levels of self-reported walking-cycling at 30 weeks gestation was associated with higher offspring BMI and diastolic blood pressure, lower high density lipoprotein cholesterol, and (in males only) higher systolic blood pressure and waist circumference. The associations were weak and of small magnitude. No associations were seen for the other aspects of physical activity assessed in the analysis. It is therefore possible that the associations observed in this study occurred due to chance. However, the authors' conclusions are in line with our own, in that there was no evidence to support the a priori hypothesised potential benefit of physical activity during pregnancy for offspring cardiovascular health.

The main strengths of our study are the large sample size, prospective nature of data collection, and the detailed measurement of a wide range of offspring cardiovascular risk factors in adolescence. The measure of maternal physical activity during pregnancy uses a weighted index, which attempts to measure the energy

For peer review only - http://bmjopen.bmj.com/site/about/guidelines.xhtml

expenditure of individuals using previously published average values for different types of physical activity (see Supplemental Table 1 in Online Resource). We were able to assess the extent to which our observed associations are confounded by maternal pre-pregnancy BMI and socio-economic factors, and explore a number of potential a priori specified mediating pathways (intrauterine factors and shared familial environment, represented by the child's own physical activity). Additionally, we used multivariate multiple imputation to examine and correct for potential bias introduced by missing data.

The results using complete case data and using multivariate multiply imputed data were similar. In both analyses, the conclusion remains that there is no strong association between reported levels of physical activity during pregnancy and offspring BMI (or any other cardiovascular risk factor). Multiple imputation gives unbiased results providing the data are missing at random, i.e. assuming that the missingness is not related to the value of the observation, conditional on the variables included in the imputation model. We included in the imputation models a wide range of variables predictive of both missingness in the outcomes and of levels of cardiovascular risk factors in those with observed data, including socio-economic factors, maternal and family characteristics, and earlier measures of the offspring's cardiovascular disease risk factors, in order to make sure that this assumption holds. The loss to follow-up in our study would result in selection bias if the association between maternal physical activity during pregnancy and offspring cardiovascular risk differed between those included in our analyses and those lost to follow-up. However, various studies have investigated the potential effects of loss to follow-up on associations in epidemiological studies, and have found that they are generally robust to even severe loss to follow-up [25-28].

A limitation of our work is that the data on maternal physical activity during pregnancy were self-reported, and thus measurement error is inevitable. It is possible that the women may under- or over- report their physical activity levels but we do not have objective data with which to evaluate this for our participants [20]. However, associations of socioeconomic position, pre-pregnancy BMI, maternal education and objectively measured offspring physical activity at age 14 were as expected, providing some evidence of face validity. MET scores may not accurately reflect the energy expenditure of physical activity in pregnancy, as they are not derived from studies of pregnant individuals. However, this is inconsequential as we use this measure as an activity classification scheme rather than an exact quantification of energy expenditure [29], and MET scores have been used in this way in several previous studies [30 31]. The "other" activity category was selected by 9.45% of the women in our study and it is possible this may reduce the strength of the associations, as several activity types with different energy expenditures are assigned the same MET score. We cannot assess women's

For peer review only - http://bmjopen.bmj.com/site/about/guidelines.xhtml

physical activity levels before pregnancy, or the extent to which women changed their physical activity levels during pregnancy, both before and after the 18 week questionnaire, as this measure of physical activity was only captured once in this cohort. Previous research with this cohort has shown that two thirds of women report at 18 weeks gestation that they have reduced their physical activity levels since becoming pregnant, but at 18 and 32 weeks gestation similar proportions of women report at least 3 hours per week of strenuous physical activity (defined as ‘sufficient to cause sweating’) [22]. Thus it is possible that physical activity either earlier or later in pregnancy may be a more important exposure for offspring cardiovascular risk – further studies are necessary to test this hypothesis.

Although we demonstrated no evidence of association between physical activity during pregnancy and offspring cardiovascular risk factors, we did find that greater levels of physical activity during pregnancy were associated with greater levels of objectively assessed physical activity in the offspring at mean age 14 years. This measure of offspring physical activity has been shown to be associated with fat mass and cardiovascular risk factors [8 32].

Our results, in line with the only existing similar study to date [17], suggest that maternal physical activity in pregnancy is unlikely to be an important determinant of later offspring cardiovascular health, though further large prospective studies of this association are needed to be confident that this is the case, particularly with studies using objectively measured physical activity at various times throughout pregnancy.

For peer review only

For peer review only - http://bmjopen.bmj.com/site/about/guidelines.xhtml

FOOTNOTES

Contributors

Louise A C Millard: Ms. Millard contributed to the design of the study, performed the analyses, drafted the initial manuscript, reviewed and revised the manuscript, and approved the final manuscript as submitted. Debbie A Lawlor: Professor Lawlor conceptualised and designed the study, critically reviewed and revised the manuscript, and approved the final manuscript as submitted. Abigail Fraser: Dr Fraser contributed to the design of the study, critically reviewed and revised the manuscript, and approved the final manuscript as submitted. Laura D Howe: Dr Howe contributed to the design of the study, supervised the analyses, critically reviewed and revised the manuscript, and approved the final manuscript as submitted.

Funding

LACM is funded by a studentship from the UK Medical Research Council. LDH was funded by a UK Medical Research Council Population Health Scientist Fellowship (G1002375). AF is funded by a UK Medical Research Council research fellowship (grant 0701594). The UK Medical Research Council (grant G074882), the Wellcome Trust (grant WT076467), and the University of Bristol provide core funding support for ALSPAC. The UK Medical Research Council (grant G0600705) and the University of Bristol provide core funding for the Medical Research Council Centre for Causal Analyses in Translational Epidemiology.

Ethics approval

ALSPAC Law and Ethics Committee and the Local Research Ethics Committees.

Data sharing statement

The ALSPAC policy on data sharing is available on the website: http://www.bristol.ac.uk/alspac. To discuss access to ALSPAC data, please contact the ALSPAC executive team on alspac-exec@bristol.ac.uk.

For peer review only

TABLES

Table 1 Association of participant characteristics with maternal physical activity during pregnancy: using multivariate multiply imputed data (N = 4,665) of individuals with data for maternal physical activity (exposure) and at least one outcome

Potential confounder	Confounder category	Standard deviation (SD) change of maternal physical activity during pregnancy associated with a 1 SD increase of confounder, with 95% confidence interval
Household social class [a]	Class I (professional)	Reference
	Class II	0.00 (-0.08 to 0.09)
	Class III non-manual	-0.18 (-0.27 to -0.09)
	Class III manual	-0.16 (-0.28 to -0.04)
	Class IV/V (manual)	-0.15 (-0.31 to 0.01)
Maternal education [b]	Less than O-level	Reference
	O-level	0.17 (0.09 to 0.25)
	A-level	0.39 (0.30 to 0.47)
	Degree or above	0.40 (0.30 to 0.49)
Ethnicity [c]	Non-white	Reference
	White	-0.11 (-0.25 to 0.03)
Parity [d]	0	Reference
	1	-0.05 (-0.11 to 0.02)
	2 +	-0.10 (-0.19 to -0.02)
Maternal smoking in pregnancy	No	Reference
	Yes	-0.09 (-0.17 to -0.01)
Previous hypertension	No	Reference
	Yes	-0.03 (-0.17 to 0.12)
Maternal pre-pregnancy BMI (SD)		-0.39 (-0.58 to -0.20)
Gestational weight gain 0-18 weeks (SD)		-0.00 (-0.03 to 0.03)

[a] Household social class: The mother recorded the occupation of both herself and her partner in a questionnaire at 32 weeks gestation, which were used to allocate them to social class groups using the 1991 Office of Population, Censuses and Surveys classification; the highest class of the mother and her partner was used in analysis
[b] Maternal education: O-level (ordinary level) exams are taken in different subjects usually at age 15–16 at the completion of legally required school attendance, equivalent to today's General Certificate of Secondary Education); A-level (Advanced-level) exams are taken in different subjects usually at age 18)
[c] Ethnicity: Mixed was recoded as non-white due to the small numbers in the mixed category (n=3)
[d] Parity: Obtained from obstetric records. Mothers with parity of two or more were grouped into a single category

For peer review only

For peer review only - http://bmjopen.bmj.com/site/about/guidelines.xhtml

Table 2 Association between maternal physical activity during pregnancy and offspring cardiovascular risk factors: using multivariate multiply imputed data (N = 4,665) of individuals with data for maternal physical activity (exposure) and at least one outcome

	**Change per 1 SD greater maternal metabolic equivalent physical activity (95% confidence intervals)**		
**Outcome**	**Minimally adjusted**	**Confounder adjusted**	**All confounders and mediators**
**Mean difference (in units specified in first column) per 1 standard deviation (SD) greater maternal physical activity during pregnancy**			
BMI, kg/m^2	-0.089 (-0.190 to 0.011)	0.004 (-0.091 to 0.098)	0.023 (-0.071 to 0.117)
Waist circumference, cm	-0.271 (-0.552 to 0.011)	-0.088 (-0.358 to 0.181)	-0.042 (-0.311 to 0.227)
SBP, mmHg	-0.044 (-0.360 to 0.272)	-0.014 (-0.332 to 0.303)	0.017 (-0.301 to 0.334)
DBP, mmHg	0.015 (-0.251 to 0.280)	0.017 (-0.251 to 0.284)	0.018 (-0.250 to 0.286)
Glucose, mmol/L	-0.013 (-0.027 to 0.001)	-0.013 (-0.027 to 0.001)	-0.012 (-0.026 to 0.001)
LDLc, mmol/L	-0.006 (-0.025 to 0.013)	-0.002 (-0.022 to 0.017)	-0.003 (-0.022 to 0.017)
HDLc, mmol/L	0.002 (-0.008 to 0.012)	0.000 (-0.010 to 0.010)	-0.001 (-0.011 to 0.009)
**Percentage difference per 1 standard deviation (SD) greater maternal physical activity during pregnancy**			
Insulin, % difference	-1.90 (-3.50 to -0.30)	-1.20 (-2.80 to 0.40)	-1.00 (-2.60 to 0.60)
Triglycerides, % difference	0.00 (-1.20 to 1.30)	0.00 (-1.30 to 1.20)	0.00 (-1.20 to 1.40)

Results of additional models with adjustment for mediators are in Supplemental Table 4 in Online Resource

Minimally adjusted: Adjusted for child gender and child age at 15 year clinic

Confounder adjusted: Adjusted for child age at 15 year clinic, child gender, maternal and family confounders (household social class, maternal education, ethnicity, parity, mother smoker, previous hypertension, maternal pre-pregnancy BMI (weight & height) and gestational weight gain 0-18 weeks)

All confounders and mediators: Confounder adjusted with additional adjustment for intrauterine mechanisms (HDP (none, gestational hypertension, preeclampsia), diabetes/glycosuria (non, existing diabetes, gestational diabetes, glycosuria), gestational weight gain 18-24 weeks, gestational weight gain 24 weeks onwards), birth weight, gestational age, and child's physical activity at age 14

For peer review only - http://bmjopen.bmj.com/site/about/guidelines.xhtml

FIGURES

Fig. 1 Flow chart of ALSPAC study participants

Fig. 2 Pathway illustration for the potential associations between physical activity during pregnancy and offspring cardiovascular risk factors

Fig. 3 Associations of maternal physical activity during pregnancy with offspring cardiovascular risk factors: using multivariate multiply imputed data (N = 4,665) of individuals with data for maternal physical activity (exposure) and at least one outcome

Footnotes for Figure 3:

Mean standard deviation (SD) difference (and 95% confidence intervals) in offspring cardiovascular risk factors (or logged distribution for insulin and triglycerides) for a one SD increase in metabolic equivalent maternal physical activity

Minimally adjusted: Adjusted for child gender and child age at 15 year clinic

Confounder adjusted: Adjusted for child age at 15 year clinic, child gender, socio-economic position confounders (household social class, maternal education, ethnicity, parity, mother smoker, previous hypertension), maternal pre-pregnancy BMI (weight & height) and gestational weight gain 0-18 weeks

Fully adjusted with adjustment for all confounders and mediators: Confounder adjusted with additional adjustment for intrauterine mechanisms (HDP (none, gestational hypertension, preeclampsia), diabetes/glycosuria (non, existing diabetes, gestational diabetes, glycosuria), gestational weight gain 18-24 weeks, gestational weight gain 24 weeks onwards), birth weight, gestational age, and child's physical activity at age 14

For peer review only - http://bmjopen.bmj.com/site/about/guidelines.xhtml

For peer review only

REFERENCES

1. Kramer MS, McDonald SW. Aerobic exercise for women during pregnancy. Cochrane Database Syst Rev 2006;**3**:CD000180 doi: 10.1002/14651858.CD000180.pub2[published Online First: Epub Date]|.

2. ACOG Committee opinion. Number 267, January 2002: exercise during pregnancy and the postpartum period. Obstet Gynecol 2002;**99**(1):171-3 doi: S0029784401017495 [pii][published Online First: Epub Date]|.

3. Davies GA, Wolfe LA, Mottola MF, MacKinnon C. Joint SOGC/CSEP clinical practice guideline: exercise in pregnancy and the postpartum period. Can J Appl Physiol 2003;**28**(3):330-41

4. (RCOG) RCoOaG. Exercise in pregnancy. Secondary Exercise in pregnancy [HTML] January 2006 2006. http://www.rcog.org.uk/womens-health/clinical-guidance/exercise-pregnancy.

5. Physical activity guidelines for Americans. Okla Nurse 2008;**53**(4):25

6. Haskell WL, Lee IM, Pate RR, et al. Physical activity and public health: updated recommendation for adults from the American College of Sports Medicine and the American Heart Association. Circulation 2007;**116**(9):1081-93 doi: CIRCULATIONAHA.107.185649 [pii]

10.1161/CIRCULATIONAHA.107.185649[published Online First: Epub Date]|.

7. Sallis JF, Patterson TL, Buono MJ, Nader PR. Relation of cardiovascular fitness and physical activity to cardiovascular disease risk factors in children and adults. Am J Epidemiol 1988;**127**(5):933-41

8. Ekelund U, Luan JA, Sherar LB, et al. Moderate to Vigorous Physical Activity and Sedentary Time and Cardiometabolic Risk Factors in Children and Adolescents. Jama-J Am Med Assoc 2012;**307**(7):704-12

9. Jayet PY, Rimoldi SF, Stuber T, et al. Pulmonary and systemic vascular dysfunction in young offspring of mothers with preeclampsia. Circulation 2010;**122**(5):488-94

10. Lawlor DA, Macdonald-Wallis C, Fraser A, et al. Cardiovascular biomarkers and vascular function during childhood in the offspring of mothers with hypertensive disorders of pregnancy: findings from the Avon Longitudinal Study of Parents and Children. Eur Heart J 2011 doi: 10.1093/eurheartj/ehr300[published Online First: Epub Date]|.

11. Lawlor DA, Fraser A, Lindsay RS, et al. Association of existing diabetes, gestational diabetes and glycosuria in pregnancy with macrosomia and offspring body mass index, waist and fat mass in later childhood: findings from a prospective pregnancy cohort. Diabetologia 2010;**53**(1):89-97 doi: 10.1007/s00125-009-1560-z[published Online First: Epub Date]|.

12. Dabelea D, Hanson RL, Lindsay RS, et al. Intrauterine exposure to diabetes conveys risks for type 2 diabetes and obesity: a study of discordant sibships. Diabetes 2000;**49**(12):2208-11

13. Clapp JF, 3rd. Morphometric and neurodevelopmental outcome at age five years of the offspring of women who continued to exercise regularly throughout pregnancy. The Journal of pediatrics 1996;**129**(6):856-63

14. Clapp JF, 3rd, Simonian S, Lopez B, Appleby-Wineberg S, Harcar-Sevcik R. The one-year morphometric and neurodevelopmental outcome of the offspring of women who continued to exercise regularly throughout pregnancy. American journal of obstetrics and gynecology 1998;**178**(3):594-9

15. Pomeroy J, Renstr√∂m F, Gradmark AM, et al. Maternal Physical Activity and Insulin Action in Pregnancy and Their Relationships With Infant Body Composition. Diabetes Care 2012 doi: 10.2337/dc12-0885[published Online First: Epub Date]|.

16. Camilla Schou Andersen MJ, Michael Gamborg, Thorkild I. A. Sørensen, and Ellen Aagaard Nohr. Maternal Recreational Exercise during Pregnancy in relation to Children's BMI at 7 Years of Age. International Journal of Pediatrics 2012;**2012**:8 doi: 10.1155/2012/920583[published Online First: Epub Date]|.

17. Danielsen I, Granstrom C, Rytter D, et al. Does physical activity during pregnancy adversely influence markers of the metabolic syndrome in adult offspring? A prospective study over two decades. J Epidemiol Community Health 2013;**67**(8):648-54 doi: 10.1136/jech-2012-202017

For peer review only - http://bmjopen.bmj.com/site/about/guidelines.xhtml

jech-2012-202017 [pii][published Online First: Epub Date]|.
18. Boyd A, Golding J, Macleod J, et al. Cohort Profile: the 'children of the 90s'--the index offspring of the Avon Longitudinal Study of Parents and Children. International journal of epidemiology 2013;**42**(1):111-27 doi: 10.1093/ije/dys064

dys064 [pii][published Online First: Epub Date]|.
19. Fraser A, Macdonald-Wallis C, Tilling K, et al. Cohort Profile: the Avon Longitudinal Study of Parents and Children: ALSPAC mothers cohort. International journal of epidemiology 2013;**42**(1):97-110 doi: 10.1093/ije/dys066

dys066 [pii][published Online First: Epub Date]|.
20. Wildschut HI, Harker LM, Riddoch CJ. The potential value of a short self-completion questionnaire for the assessment of habitual physical activity in pregnancy. J Psychosom Obstet Gynaecol 1993;**14**(1):17-29
21. Ainsworth BE, Haskell WL, SD H, et al. Compendium of Physical Activities: a second update of codes and MET values. Med Sci Sports Exerc 2011
22. Liu J, Blair SN, Teng Y, Ness AR, Lawlor DA, Riddoch C. Physical activity during pregnancy in a prospective cohort of British women: results from the Avon longitudinal study of parents and children. European journal of epidemiology 2011;**26**(3):237-47 doi: 10.1007/s10654-010-9538-1[published Online First: Epub Date]|.
23. Stata Statistical Software: Release 11 [program], 2009.
24. Clinical Guidelines on the Identification, Evaluation, and Treatment of Overweight and Obesity in Adults--The Evidence Report. National Institutes of Health. Obes Res 1998;**6 Suppl 2**:51S-209S
25. Howe LD, Tilling K, Galobardes B, Lawlor DA. Loss to follow-up in cohort studies: bias in estimates of socioeconomic inequalities. Epidemiology 2013;**24**(1):1-9 doi: 10.1097/EDE.0b013e31827623b1[published Online First: Epub Date]|.
26. Nilsen RM, Vollset SE, Gjessing HK, et al. Self-selection and bias in a large prospective pregnancy cohort in Norway. Paediatr Perinat Epidemiol 2009;**23**(6):597-608 doi: 10.1111/j.1365-3016.2009.01062.x

PPE1062 [pii][published Online First: Epub Date]|.
27. Nohr EA, Frydenberg M, Henriksen TB, Olsen J. Does low participation in cohort studies induce bias? Epidemiology 2006;**17**(4):413-18
28. Pizzi C, De Stavola BL, Pearce N, et al. Selection bias and patterns of confounding in cohort studies: the case of the NINFEA web-based birth cohort. J Epidemiol Community Health 2012;**66**(11):976-81 doi: 10.1136/jech-2011-200065

jech-2011-200065 [pii][published Online First: Epub Date]|.
29. Ainsworth BE, Haskell WL, Whitt MC, et al. Compendium of physical activities: an update of activity codes and MET intensities. Med Sci Sports Exerc 2000;**32**(9 Suppl):S498-504
30. Borodulin KM, Evenson KR, Wen F, Herring AH, Benson AM. Physical activity patterns during pregnancy. Med Sci Sports Exerc 2008;**40**(11):1901-8 doi: 10.1249/MSS.0b013e31817f1957[published Online First: Epub Date]|.
31. Rousham EK, Clarke PE, Gross H. Significant changes in physical activity among pregnant women in the UK as assessed by accelerometry and self-reported activity. Eur J Clin Nutr 2006;**60**(3):393-400
32. Riddoch CJ, Leary SD, Ness AR, et al. Prospective associations between objective measures of physical activity and fat mass in 12-14 year old children: the Avon Longitudinal Study of Parents and Children (ALSPAC). Brit Med J 2009;**339** doi: ARTN b4544

DOI 10.1136/bmj.b4544[published Online First: Epub Date]|.

For peer review only - http://bmjopen.bmj.com/site/about/guidelines.xhtml

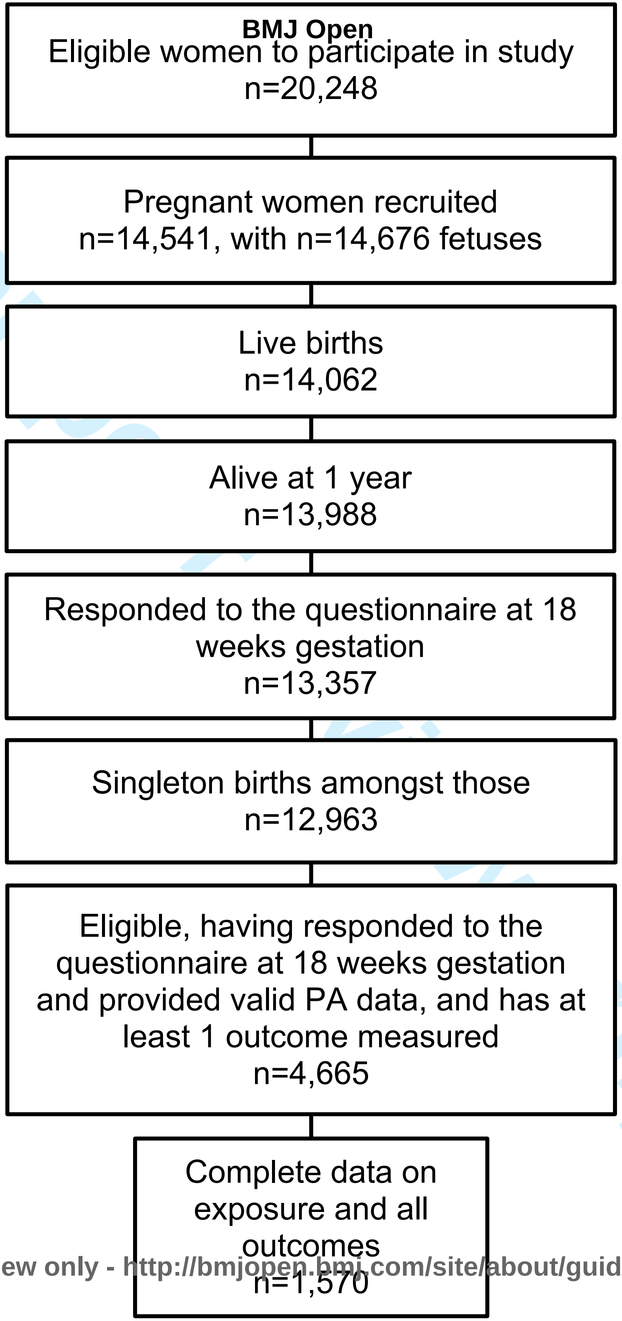

For peer review only - http://bmjopen.bmj.com/site/about/guidelines.xhtml

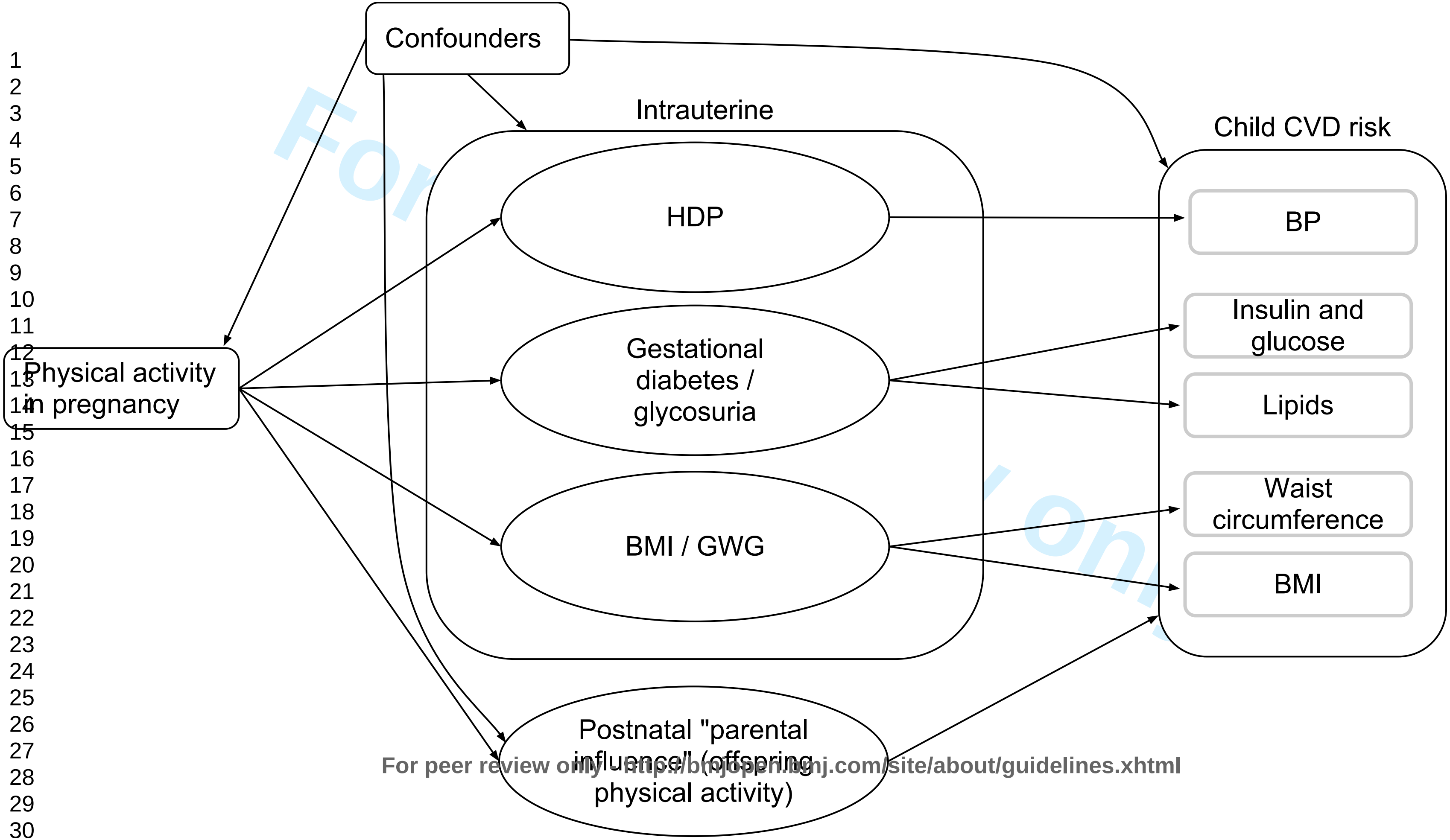

For peer review only - http://bmjopen.bmj.com/site/about/guidelines.xhtml

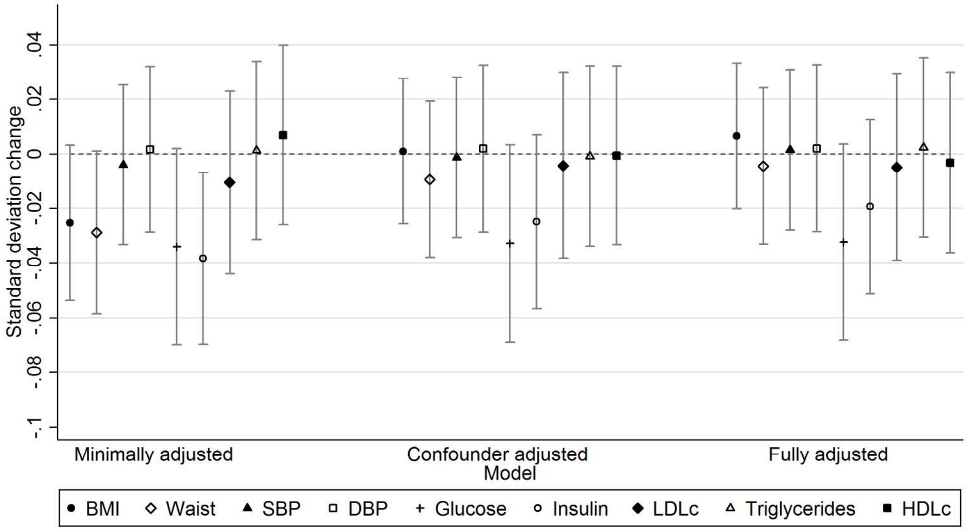

846x476mm (72 x 72 DPI)

review only

Physical activity during pregnancy and offspring cardiovascular risk factors: findings from a prospective cohort study

Supplementary Material

Louise A C Millard, MSc[1,2*], Debbie A Lawlor, PhD [1,2], Abigail Fraser, PhD [1,2], Laura D Howe, PhD[1,2]

1 – MRC Integrative Epidemiology Unit at the University of Bristol,
2 – School of Social and Community Medicine, University of Bristol

*Corresponding author.
Correspondence to: LAC Millard, School of Social and Community Medicine, University of Bristol, Oakfield House, Bristol, UK, BS8 2BN, [louise.millard@bristol.ac.uk], tel +44 117 331 0084, fax: +44 117 331 0123

For peer review only

For peer review only - http://bmjopen.bmj.com/site/about/guidelines.xhtml

SUPPLEMENTARY METHODS

Measures used in this study

Cardiovascular risk factors in the offspring ~~Weight and height were measured with the child in light clothing and without shoes. Weight was measured to the nearest 0.1 kg with Tanita scales and height to the nearest 0.1cm with a Harpenden stadiometer. BMI was calculated as weight in kilograms divided by height in metres squared. A flexible tape was used to measure waist circumference to the nearest 1 mm at the mid-point between the lower ribs and the pelvic bone. Blood pressure was measured with a Dinamap 9301 Vital Signs Monitor (Morton Medical, London) with the correct cuff size. Two readings of systolic and diastolic blood pressure were recorded, with the child at rest and their arm supported, and we used the mean of the two measures.~~
Clinic participants were asked to fast overnight for those attending in the morning or for a minimum of six hours for those attending after lunch. Blood samples were immediately spun and frozen at −80°C. Measurements were assayed three to nine months after samples were taken, with no previous freeze-thaw cycles. Plasma lipid concentrations (total cholesterol, triglycerides, and high density lipoprotein cholesterol (HDLc) were measured by modification of the standard Lipid Research Clinics Protocol by using enzymatic reagents for lipid determination. Low density lipoprotein cholesterol (LDLc) concentrations were determined from these with the Friedwald equation (LDLc=total cholesterol−HDLc+triglycerides×0.45 mmol/l)). ~~Insulin was measured with an enzyme linked immunosorbent assay (ELISA) (Mercodia, Uppsala, Sweden) that does not cross react with proinsulin, and plasma glucose was measured with an automated assay.~~

Assessment of potential mediators

Hypertensive disorder of pregnancy (HDP) was assessed using data abstracted from obstetric records that included every measurement of systolic blood pressure (SBP), diastolic blood pressure (DBP), and proteinuria recorded and the corresponding gestational age at the time of these measurements. We applied the International Society for the Study of Hypertension in Pregnancy [1] criteria to all of the clinic data in order to determine women with pre-eclampsia and those with gestational hypertension, and categorized the mothers into one of three mutually exclusive categories; no HDP, gestational hypertension, or pre-eclampsia.

Gestational weight gain was calculated from a linear spline multilevel model developed using all available pregnancy weight measurements (see [2] for further details). We use 3 gestational weight gain variables; 0-18 weeks, 18-28 weeks, 28 weeks onwards, where the 0-18 week variable is used as a confounder (see below) because it refers to the time before the exposure measurement (18 weeks antenatal) whereas the gestational weight gain variables from 18 weeks gestation onwards are used as potential mediators because they refer to the time between exposure and outcome measurement.

At recruitment, women were asked about existing diabetes and any previous history of gestational diabetes. Research midwives abstracted information on clinical diagnoses of gestational diabetes and glycosuria from the antenatal, pregnancy and postnatal medical records using a standard protocol. Using these data, we classified the women into one of four mutually exclusive categories: no evidence of glycosuria or diabetes; existing diabetes before the pregnancy; gestational diabetes (i.e. a diagnosis in the medical records of gestational diabetes in any woman with no history of existing diabetes); and glycosuria (i.e. ++ glycosuria on two occasions in women with no evidence of existing diabetes or gestational diabetes).

Child's physical activity was measured using an accelerometer (MTI Actigraph AM7164 2.2) worn over a period of 7 days. The accelerometer is worn around the waist and detects acceleration and deceleration in a vertical plane as a combined function of movement frequency and intensity. A measure of time spent in moderate and vigorous physical activity (MVPA, equivalent to brisk walking or running) was calculated and averaged over valid days. The accelerometer must have been worn for at least 10 hours a day for at least three days after deletion of missing data to be considered valid [3-5]. To maximize our sample size for this analysis, we used a multilevel model to predict MVPA at exactly 14 years for all participants who had accelerometer measures from at least one of the following time points: age 11, age 13, and age 15. The multilevel model (two levels: occasion and individual) uses all available data from all eligible participants (those with one or more measure) under a missing at random assumption to predict MVPA for all participants, assuming linear rates of change between 11-13 and 13-15 years. Although the model generates values of predicted MVPA between 11 and 15 years old, we included only predicted MVPA at 14 years in our model since there was a high correlation between predicted levels of MVPA at all ages. MLwiN [6] and the runmlwin [7] Stata command were used for the multilevel model.

Gestational age and infant birthweight were recorded in the delivery room and abstracted from obstetric records and/or birth notifications.

For peer review only

Potential confounders

Maternal weight and height At enrolment (questionnaire at approximately 12 weeks gestation), the mother was asked to record her height and pre-pregnancy weight. Maternal pre pregnancy body mass index (BMI) was calculated by dividing her weight in kilograms by her height in meters squared.

Child age & gender Child age was recorded in months as they arrived for the age 15 year clinic. Infant sex was recorded in the delivery room or abstracted from obstetric records or birth notifications.

Household social class The mother recorded the occupation of both herself and her partner in a questionnaire at 32 weeks gestation, which were used to allocate them to social class groups (I, II, III manual, III non-manual, IV, V) using the 1991 Office of Population, Censuses and Surveys classification; the highest class of the mother and her partner was used in analysis.

Maternal education A questionnaire at 32 weeks gestation asked mothers to report their educational attainment. Maternal educational attainment was categorized as below O-level (ordinary level; exams taken in different subjects usually at age 15–16 at the completion of legally required school attendance, equivalent to today's General Certificate of Secondary Education), O-level only, A-level (advanced-level; exams taken in different subjects usually at age 18), or university degree or above.

Ethnicity A binary variable denoting offspring white or non-white ethnicity was used, where mixed was recoded as non-white due to the small numbers in the mixed category (n=3).

Parity Parity was obtained from obstetric records. Mothers with parity of two or more were grouped into a single category.

Maternal smoking during pregnancy The mother was asked about her smoking habits on two occasions during pregnancy (the 18 week and 32 week antenatal questionnaires), and a dichotomous variable was created for any smoking during pregnancy.

Previous hypertension Mothers were asked about their history of high blood pressure with a yes/no question in a self-administered questionnaire completed at 12 weeks gestation.

Missing data

We used multivariate multiple imputation in Stata as described by Royston [8]. We carried out 20 cycles of regression switching and generated 20 imputation data sets. All variables included in our analyses were used to impute the missing data. The multiple multivariate imputation approach creates a number of copies of the data (in this case, we generated 20 copies) in which missing values are imputed by chained equations [8]. The main analysis results are obtained by averaging across the results from each of these 20 data sets using Rubin's rules, which ensure that the standard errors for any regression coefficients take account of uncertainty in the imputations as well as uncertainty in the estimation [8].

To inform the imputation we included additional variables: cardiovascular disease (CVD) risk factors (BMI and waist circumference at age 11, and SBP, DBP, low density lipoprotein cholesterol (LDLc), high density lipoprotein cholesterol (HDLc), triglycerides at age 9), physical activity at age 12 and measures predictive of missingness of CVD risk factors (paternal education, child total fat mass (age 9), paternal height, maternal age at delivery and birth length). The following variables were log transformed to produce distributions that were approximately normally to improve the imputation: child BMI, waist circumference, LDLc, HDLc and triglycerides age 15, physical activity age 12 and 14, total fat mass and triglycerides age 9, BMI and waist circumference age 11 and maternal BMI.

For peer review only

SUPPLEMENTARY RESULTS

Supplemental Table 1 Physical activity MET scores

Activity	MET score	Number of hours equating to mean MET score [a]
Jogging	7.0	2.3
Brisk walking	3.8	4.2
Swimming	6.0	2.6
Antenatal exercise	2.0	7.9
Keep fit exercise	3.5	4.5
Cycling	5.5	2.9
Aerobics	6.5	2.4
Tennis	4.5	3.5
Yoga	2.5	6.3
Weight training	3.0	5.3
Squash	12.0	1.3
Other	3.5	4.5

[a] Average MET score for participants included in our analyses = 15.8. We have calculated what this equates to in terms of each activity, by dividing 15.8 by the MET score for each activity. ~~The number of hours equating to the mean MET score is calculated as: 15.8 / MET score.~~ Thius this column represents the average number of hours an individual would spend to reach the mean MET score solely through partaking in each particular activity.

For peer review only

Supplemental Table 2 Summary of variable distributions for observed and imputed data

		Number of participants with non-missing data	Observed data (all individuals) (n (%) or mean (standard deviation (SD)))	Observed data (individuals with complete data, N=1,570) (n (%) or mean (SD))	Imputed data [N= 4,665] (n (%) or mean (standard error)) [c]
Parity (n, %)	0	4633	2259 (48.8)	793 (50.5)	2277 (48.8)
	1		1598 (34.5)	553 (35.2)	1608 (34.5)
	2+		776 (16.8)	224 (14.3)	781 (16.7)
Household	i	4466	761 (17.0)	296 (18.9)	777 (16.7)
social class	ii		2058 (46.1)	766 (48.8)	2123 (45.5)
(n)	iiin		1074 (24.1)	354 (22.5)	1134 (24.3)
	iiim		400 (9.0)	108 (6.9)	438 (9.4)
	iv		173 (3.9)	46 (2.9)	194 (4.2)
Mothers	< o-level	4559	875 (19.2)	241 (15.4)	909 (19.5)
Education	o-level		1595 (35.0)	524 (33.4)	1633 (35.0)
(n)	a-level		1292 (28.3)	486 (31.0)	1315 (28.2)
	degree or above		797 (17.5)	319 (20.3)	808 (17.3)
Child Ethnicity white (n)		4649	4450 (95.7)	1516 (96.6)	4465 (95.7)
Maternal pre-pregnancy weight, kg (mean, SD)		4350	60.56 (11.91)	59.90 (11.18)	60.48 (0.18)
Maternal pre-pregnancy height, cm (mean)		4497	164.33 (6.68)	164.44 (6.72)	164.30 (0.10)
Maternal gestational weight gain 0 – 18 weeks, kg/week (mean)		4350	0.31 (0.17)	0.32 (0.17)	0.31 (0.00)
Mother previous hypertension, yes (n)		4087	190 (4.7)	60 (3.8)	216 (4.6)
Mother smokes in pregnancy, yes (n)		4653	762 (16.4)	238 (15.2)	764 (16.4)
Child gender, female (n)		4665	2436 (52.2)	801 (51.0)	2436 (52.2)
HDP (n)	None	4644	3885 (83.7)	1352 (86.1)	3903 (83.7)
	Pre-eclampsia		89 (1.9)	20 (1.3)	90 (1.9)
	Gestational hypertension		670 (14.4)	198 (12.6)	673 (14.4)
Maternal	None	4548	4358 (95.8)	1515 (96.5)	4468 (95.8)
diabetes	Existing diabetes		20 (0.4)	5 (0.3)	22 (0.5)
(n)	Gestational diabetes		27 (0.6)	8 (0.5)	28 (0.6)
	Glycosuria		143 (3.1)	42 (2.7)	147 (3.2)
Maternal physical activity, MET hours/week (mean) [a]		4665	16.41 (15.32)	17.02 (15.87)	16.41 (0.22)
Child BMI age 15, kg/m^2 (mean)		4603	21.44 (3.53)	21.19 (3.13)	21.45 (0.05)

For peer review only - http://bmjopen.bmj.com/site/about/guidelines.xhtml

Child waist circumference age 15, cm (mean)	3828	76.67 (8.92)	76.54 (8.58)	77.15 (0.14)
Child SBP age 15, mmHg (mean)	4345	123.21 (10.84)	123.52 (10.88)	123.28 (0.17)
Child DBP age 15, mmHg (mean)	4345	67.63 (8.74)	66.63 (8.45)	67.64 (0.13)
Child glucose age 15, mmol/L (mean)	2957	5.21 (0.39)	5.20 (0.37)	5.21 (0.01)
Child insulin age 15, IU/L (mean)	2953	10.32 (6.50)	9.60 (4.74)	10.34 (0.11)
Child LDLc age 15, mmol/L (mean)	2957	2.09 (0.56)	2.09 (0.54)	2.09 (0.01)
Child triglycerides age 15, mmol/L (mean)	2957	0.84 (0.39)	0.81 (0.33)	0.84 (0.01)
Child HDLc age 15, mmol/L (mean)	2957	1.28 (0.29)	1.30 (0.29)	1.29 (0.01)
Child age at 15 year clinic, weeks (mean)	4665	808.22 (17.45)	805.00 (12.49)	808.22 (0.26)
Child physical activity age 14 (mean) [b]	3938	1.45 (0.62)	1.48 (0.63)	1.45 (0.01)
Maternal gestational weight gain 18 - 28/40, kg/week (mean)	4350	0.54 (0.17)	0.53 (0.16)	0.53 (0.00)
Maternal gestational weight gain 28/40 onwards kg/week (mean)	4350	0.47 (0.19)	0.46 (0.19)	0.47 (0.00)
Birthweight, grams (mean)	4603	3440.06 (523.94)	3497.28 (472.43)	3439.64 (7.69)
Gestational age, weeks (mean)	4494	32.50 (1.21)	32.51 (1.12)	32.50 (0.02)

[a] Metabolic equivalent maternal physical activity
[b] Mean daily minutes of MVPA (predicted)
[c] Multivariate multiply imputed data over 20 imputation data sets of all mother-offspring pairs with a value of physical activity during pregnancy (exposure) and at least one offspring cardiovascular risk factor age 15.5 (outcomes)

For peer review only

Supplemental Table 3 Association of maternal physical activity with potential mediators of the relationship between physical activity during pregnancy and offspring cardiovascular risk factors: using multivariate multiply imputed data (N = 4,665) of individuals with data for maternal physical activity (exposure) and at least one outcome

Potential mediator	Mediator category	Change per 1 SD greater maternal metabolic equivalent physical activity during pregnancy
**Odds ratio per SD greater maternal physical activity during pregnancy, null value = 1**		
Hypertensive Disorders of Pregnancy	None	Reference
	Pre-eclampsia	-0.04 (-0.25 to 0.17)
	Gestational hypertension	-0.04 (-0.12 to 0.05)
Diabetes	None	Reference
	Existing diabetes	0.18 (-0.23 to 0.58)
	Gestational diabetes	-0.29 (-0.73 to 0.15)
	Glycosuria	-0.03 (-0.20 to 0.14)
**Mean difference per SD greater maternal physical activity during pregnancy, null value = 0**		
Gestational weight gain (SD)	18 - 28 weeks	-0.02 (-0.05 to 0.01)
	28 weeks onwards	-0.01 (-0.04 to 0.02)
Gestational age (SD)		-0.02 (-0.05 to 0.01)
Birthweight (SD)		-0.01 (-0.04 to 0.02)
Offspring moderate to vigorous physical activity age 14 (SD)		0.06 (0.03 to 0.09)

SD: Standard deviation

For peer review only - http://bmjopen.bmj.com/site/about/guidelines.xhtml

Supplemental Table 4 Association between maternal physical activity during pregnancy and offspring cardiovascular risk factors: using multivariate multiply imputed data (N = 4,665) of individuals with data for maternal physical activity (exposure) and at least one outcome

	Change per 1 standard deviation (SD) greater maternal metabolic equivalent physical activity (95% confidence intervals)					
Outcome	Minimal adjustment for child age and gender	Adjusted for confounders: household social class, maternal education, ethnicity, parity, maternal smoking during pregnancy, previous hypertension, maternal pre-pregnancy BMI and gestational weight gain 0-18 weeks	Adjusted for confounders and gestational diabetes, HDP, gestational weight gain	Adjusted for confounders, gestational diabetes, HDP, gestational weight gain, gestational age, and birthweight	Adjusted for confounders and offspring physical activity	Adjusted for all confounders and mediators
**Mean difference (in units specified in first column) per 1 SD greater maternal physical activity during pregnancy**						
BMI, kg/m^2	-0.089 (-0.190 to 0.011)	0.004 (-0.091 to 0.098)	0.004 (-0.090 to 0.099)	0.006 (-0.088 to 0.100)	0.021 (-0.073 to 0.115)	0.023 (-0.071 to 0.117)
Waist circumference, cm	-0.271 (-0.552 to 0.011)	-0.088 (-0.358 to 0.181)	-0.085 (-0.354 to 0.184)	-0.082 (-0.351 to 0.186)	-0.048 (-0.317 to 0.221)	-0.042 (-0.311 to 0.227)
SBP, mmHg	-0.044 (-0.360 to 0.272)	-0.014 (-0.332 to 0.303)	-0.012 (-0.329 to 0.305)	-0.008 (-0.325 to 0.309)	0.012 (-0.307 to 0.330)	0.017 (-0.301 to 0.334)
DBP, mmHg	0.015 (-0.251 to 0.280)	0.017 (-0.251 to 0.284)	0.019 (-0.248 to 0.287)	0.022 (-0.246 to 0.290)	0.014 (-0.254 to 0.282)	0.018 (-0.250 to 0.286)
Glucose, mmol/L	-0.013 (-0.027 to 0.001)	-0.013 (-0.027 to 0.001)	-0.013 (-0.026 to 0.001)	-0.013 (-0.027 to 0.001)	-0.013 (-0.026 to 0.001)	-0.012 (-0.026 to 0.001)
LDLc, mmol/L	-0.006 (-0.025 to 0.013)	-0.002 (-0.022 to 0.017)	-0.003 (-0.022 to 0.017)	-0.003 (-0.022 to 0.017)	-0.002 (-0.022 to 0.017)	-0.003 (-0.022 to 0.017)
HDLc, mmol/L	0.002 (-0.008 to 0.012)	0.000 (-0.010 to 0.010)	0.000 (-0.010 to 0.010)	0.000 (-0.010 to 0.010)	-0.001 (-0.011 to 0.009)	-0.001 (-0.011 to 0.009)
**Percentage difference per 1 SD greater maternal physical activity during pregnancy**						
Insulin, % difference	-1.90 (-3.50 to -0.30)	-1.20 (-2.80 to 0.40)	-1.20 (-2.80 to 0.40)	-1.20 (-2.80 to 0.40)	-1.00 (-2.60 to 0.60)	-1.00 (-2.60 to 0.60)
Triglycerides, %	0.00 (-1.20 to 1.30)	0.00 (-1.30 to 1.20)	0.00 (-1.30 to 1.30)	0.00 (-1.30 to 1.30)	0.00 (-1.20 to 1.30)	0.00 (-1.20 to 1.40)

For peer review only - http://bmjopen.bmj.com/site/about/guidelines.xhtml

difference						

For peer review only

Supplemental Fig. 1 BMI interaction analysis showing associations of maternal physical activity with child offspring CVD outcomes for overweight and not overweight individuals using multiply imputed sample (N=4,665)

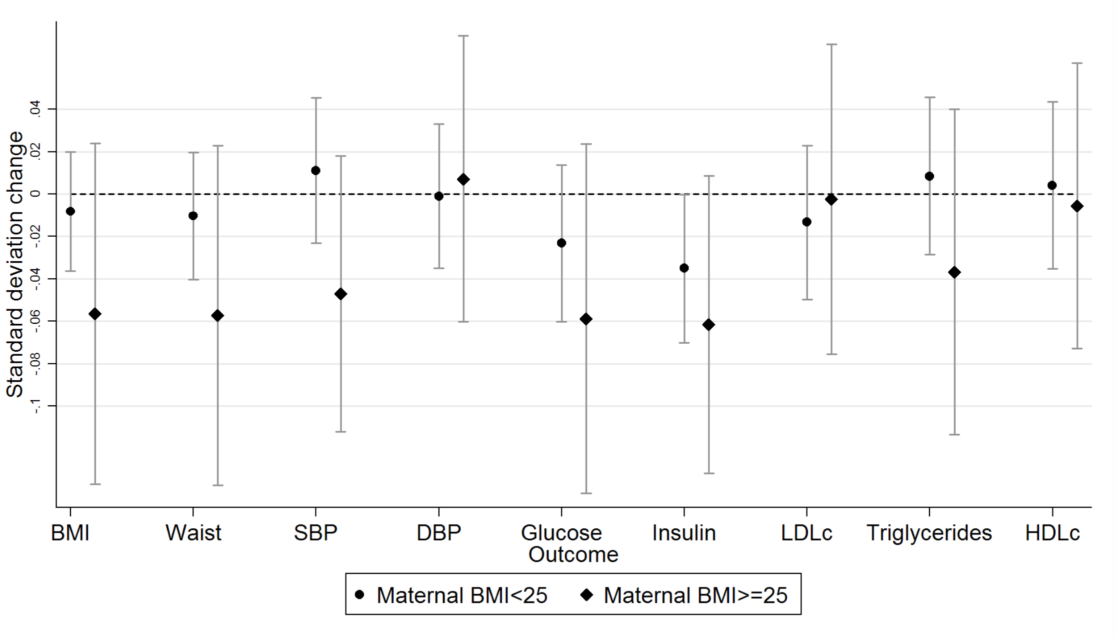

Mean standard deviation difference (and 95% confidence intervals) in offspring cardiovascular risk factors (or logged distribution for insulin and triglycerides) for a 1 standard deviation increase in metabolic equivalent maternal physical activity during pregnancy

review only

Supplemental Fig. 2 Associations of maternal metabolic equivalent maternal physical activity during pregnancy with child BMI using complete case and multiply imputed samples

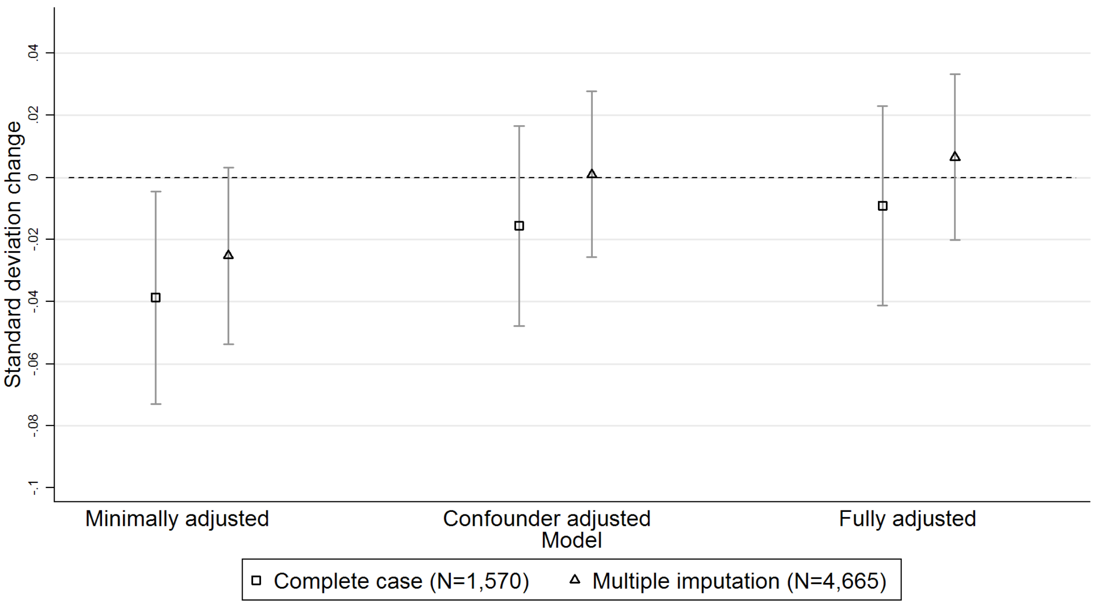

Mean standard deviation difference (and 95% confidence intervals) in offspring BMI per 1 standard deviation increase in metabolic equivalent maternal physical activity during pregnancy

Multiple imputation: 20 imputations (Stata ice command)
Multiple imputation (N=4,665): all mother-offspring pairs with a value of physical activity during pregnancy and at least one offspring cardiovascular risk factor age 15.5
Complete case: Original data excluding individuals with any missing data for all variables per outcome

For peer review only

Supplemental Fig. 3 Associations of maternal metabolic equivalent maternal physical activity during pregnancy with child blood insulin levels using complete case and multiply imputed samples

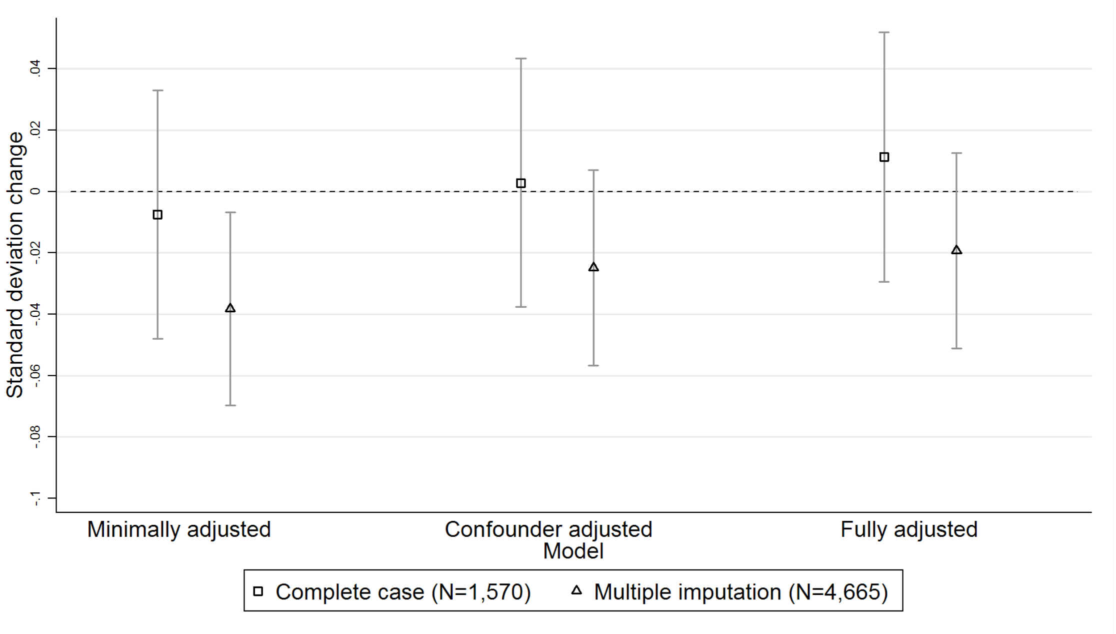

Mean standard deviation difference (and 95% confidence intervals) in log transformed offspring insulin per 1 standard deviation increase in metabolic equivalent maternal physical activity during pregnancy

Multiple imputation: 20 imputations (Stata ice command)
Multiple imputation (N=4,665): all mother-offspring pairs with a value of physical activity during pregnancy and at least one offspring cardiovascular risk factor age 15.5
Complete case: Original data excluding individuals with any missing data for all variables per outcome

review only

Supplemental Fig. 4 Associations of maternal metabolic equivalent maternal physical activity during pregnancy with child blood glucose levels using complete case and multiply imputed samples

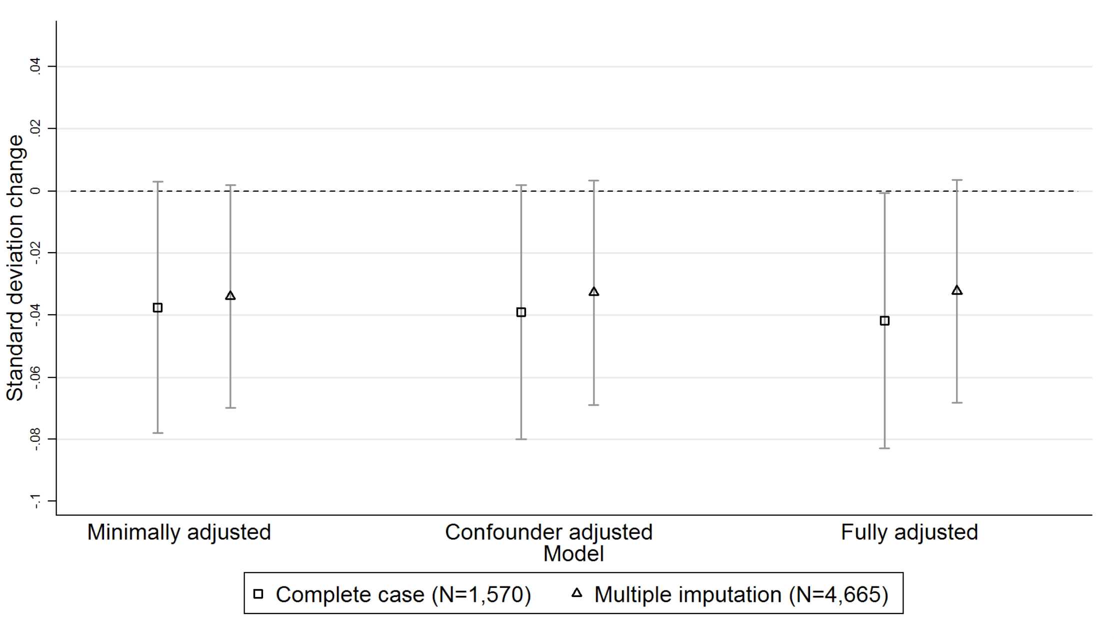

Mean standard deviation difference (and 95% confidence intervals) in offspring glucose per 1 standard deviation increase in metabolic equivalent maternal physical activity during pregnancy

Multiple imputation: 20 imputations (Stata ice command)
Multiple imputation (N=4,665): all mother-offspring pairs with a value of physical activity during pregnancy and at least one offspring cardiovascular risk factor age 15.5
Complete case: Original data excluding individuals with any missing data for all variables per outcome

For peer review only - http://bmjopen.bmj.com/site/about/guidelines.xhtml

REFERENCES

1. Davey DA, MacGillivray I. The classification and definition of the hypertensive disorders of pregnancy. American journal of obstetrics and gynecology 1988;**158**(4):892-8 doi: 0002-9378(88)90090-7 [pii][published Online First: Epub Date]|.
2. Fraser A, Tilling K, Macdonald-Wallis C, et al. Association of maternal weight gain in pregnancy with offspring obesity and metabolic and vascular traits in childhood. Circulation 2010;**121**(23):2557-64 doi: 10.1161/CIRCULATIONAHA.109.906081[published Online First: Epub Date]|.
3. Mattocks C, Leary S, Ness A, et al. Calibration of an accelerometer during free-living activities in children. Int J Pediatr Obes 2007;**2**(4):218-26 doi: 781692295 [pii]

10.1080/17477160701408809[published Online First: Epub Date]|.
4. Mattocks C, Ness A, Leary S, et al. Use of Accelerometers in a Large Field-Based Study of Children: Protocols, Design Issues, and Effects on Precision. J Phys Act Health 2008;**5**:S98-S111
5. Riddoch CJ, Mattocks C, Deere K, et al. Objective measurement of levels and patterns of physical activity. Arch Dis Child 2007;**92**(11):963-69 doi: Doi 10.1136/Adc.2006.112136[published Online First: Epub Date]|.
6. MLwiN Version 2.1, Centre for Multilevel Modelling, University of Bristol. [program]. Bristol, 2009.
7. runmlwin: Stata module for fitting multilevel models in the MLwiN software package. Centre for Multilevel Modelling, University of Bristol. [program]. Bristol, 2009.
8. Royston P. Multiple imputation of missing values. Stata J, 2004:227–41.

For peer review only

STROBE Statement—Checklist of items that should be included in reports of ***cohort studies***

	Item No	Recommendation
**Title and abstract**	1	(*a*) Indicate the study's design with a commonly used term in the title or the abstract
		(*b*) Provide in the abstract an informative and balanced summary of what was done and what was found
**Introduction**		
Background/rationale	2	Explain the scientific background and rationale for the investigation being reported
Objectives	3	State specific objectives, including any prespecified hypotheses
**Methods**		
Study design	4	Present key elements of study design early in the paper
Setting	5	Describe the setting, locations, and relevant dates, including periods of recruitment, exposure, follow-up, and data collection
Participants	6	(*a*) Give the eligibility criteria, and the sources and methods of selection of participants. Describe methods of follow-up
		(*b*) For matched studies, give matching criteria and number of exposed and unexposed
Variables	7	Clearly define all outcomes, exposures, predictors, potential confounders, and effect modifiers. Give diagnostic criteria, if applicable
Data sources/ measurement	8*	For each variable of interest, give sources of data and details of methods of assessment (measurement). Describe comparability of assessment methods if there is more than one group
Bias	9	Describe any efforts to address potential sources of bias
Study size	10	Explain how the study size was arrived at
Quantitative variables	11	Explain how quantitative variables were handled in the analyses. If applicable, describe which groupings were chosen and why
Statistical methods	12	(*a*) Describe all statistical methods, including those used to control for confounding
		(*b*) Describe any methods used to examine subgroups and interactions
		(*c*) Explain how missing data were addressed
		(*d*) If applicable, explain how loss to follow-up was addressed
		(*e*) Describe any sensitivity analyses
**Results**		
Participants	13*	(a) Report numbers of individuals at each stage of study—eg numbers potentially eligible, examined for eligibility, confirmed eligible, included in the study, completing follow-up, and analysed
		(b) Give reasons for non-participation at each stage
		(c) Consider use of a flow diagram
Descriptive data	14*	(a) Give characteristics of study participants (eg demographic, clinical, social) and information on exposures and potential confounders
		(b) Indicate number of participants with missing data for each variable of interest
		(c) Summarise follow-up time (eg, average and total amount)
Outcome data	15*	Report numbers of outcome events or summary measures over time
Main results	16	(*a*) Give unadjusted estimates and, if applicable, confounder-adjusted estimates and their precision (eg, 95% confidence interval). Make clear which confounders were adjusted for and why they were included
		(*b*) Report category boundaries when continuous variables were categorized
		(*c*) If relevant, consider translating estimates of relative risk into absolute risk for a meaningful time period

For peer review only - http://bmjopen.bmj.com/site/about/guidelines.xhtml

Other analyses	17	Report other analyses done—eg analyses of subgroups and interactions, and sensitivity analyses
**Discussion**		
Key results	18	Summarise key results with reference to study objectives
Limitations	19	Discuss limitations of the study, taking into account sources of potential bias or imprecision. Discuss both direction and magnitude of any potential bias
Interpretation	20	Give a cautious overall interpretation of results considering objectives, limitations, multiplicity of analyses, results from similar studies, and other relevant evidence
Generalisability	21	Discuss the generalisability (external validity) of the study results
**Other information**		
Funding	22	Give the source of funding and the role of the funders for the present study and, if applicable, for the original study on which the present article is based

*Give information separately for exposed and unexposed groups.

Note: An Explanation and Elaboration article discusses each checklist item and gives methodological background and published examples of transparent reporting. The STROBE checklist is best used in conjunction with this article (freely available on the Web sites of PLoS Medicine at http://www.plosmedicine.org/, Annals of Internal Medicine at http://www.annals.org/, and Epidemiology at http://www.epidem.com/). Information on the STROBE Initiative is available at http://www.strobe-statement.org.

For peer review only

Physical activity during pregnancy and offspring cardiovascular risk factors: findings from a prospective cohort study

Louise A C Millard, MSc[1,2*], Debbie A Lawlor, PhD [1,2], Abigail Fraser, PhD [1,2], Laura D Howe, PhD[1,2]

1 – MRC Integrative Epidemiology Unit at the University of Bristol,
2 – School of Social and Community Medicine, University of Bristol

*Corresponding author.
Correspondence to: LAC Millard, School of Social and Community Medicine, University of Bristol, Oakfield House, Bristol, UK, BS8 2BN, [louise.millard@bristol.ac.uk], tel +44 117 331 0084, fax: +44 117 331 0123

For peer review only

For peer review only - http://bmjopen.bmj.com/site/about/guidelines.xhtml

ABSTRACT

Objectives

The long term consequences of maternal physical activity during pregnancy for offspring cardiovascular health are unknown. We examined the association of maternal self-reported physical activity in pregnancy (18 weeks gestation) with offspring cardiovascular risk factors at age 15.

Design

Prospective cohort study.

Setting

The Avon Longitudinal Study of Parents and Children (ALSPAC).

Participants

4,665 maternal-offspring pairs (based on a sample with multivariate multiple imputation to deal with missing data) from the Avon Longitudinal Study of Parents and Children, a prospective cohort based in the South West of England with mothers recruited in pregnancy in 1991-1992.

Primary and secondary outcome measures

Offspring cardiovascular risk factors at age 15; body mass index, waist circumference, systolic blood pressure, diastolic blood pressure, glucose, insulin, low density lipoprotein cholesterol, high density lipoprotein cholesterol and triglycerides.

Results

Greater maternal physical activity was associated with lower BMI, waist circumference, glucose and insulin in unadjusted analyses. The magnitude of associations was generally small with wide confidence intervals, and most associations attenuated towards the null after adjusting for confounders. The strongest evidence of association after adjustment for confounders was for glucose, although the 95% confidence interval for this association includes the null; a one standard deviation greater physical activity during pregnancy was associated with a -0.013 mmol/l difference in offspring glucose levels (equivalent to approximately one third of a standard deviation; 95% confidence interval -0.027 to 0.001 mmol/l).

For peer review only - http://bmjopen.bmj.com/site/about/guidelines.xhtml

Conclusions

Our results suggest that maternal physical activity in pregnancy, measured at 18 weeks gestation, is unlikely to be an important determinant of later offspring cardiovascular health. There was some suggestion of association with offspring glucose, but given that all other associations (including insulin) were null after adjustment for confounders, this result should be interpreted with caution.

For peer review only

ARTICLE SUMMARY

Article focus

- To examine the association of maternal self-reported physical activity in pregnancy (18 weeks gestation) with offspring cardiovascular risk factors at age 15.

Key messages

- Self-reported pPhysical activity in pregnancy, at 18 weeks gestation, is unlikely to be an important determinant of later offspring cardiovascular health.

Strengths and limitations of this study

- The main strengths of our study are the large sample size, prospective nature of data collection, and the detailed measurement of a wide range of offspring cardiovascular risk factors in adolescence.
- A limitation of our work is that the data on maternal physical activity during pregnancy were self-reported, and thus measurement error is possible. We cannot assess women's physical activity levels before pregnancy, or the extent to which women changed their physical activity levels between the 18 week questionnaire and the end of the pregnancy. Thus it is possible that physical activity later in pregnancy may be a more important exposure for offspring cardiovascular risk.

For peer review only

INTRODUCTION

There is increasing interest in the potential effects of physical activity during pregnancy on both mother and offspring [1]. Current policy encourages physical activity during pregnancy, with UK guidelines recommending at least 30 minutes of moderate intensity activity at least 4 times a week [2-4], and US guidelines recommending 2 ½ hours each week [5]. However, the evidence regarding potential risks and benefits of physical activity during pregnancy is unclear, with a Cochrane systematic review of randomised controlled trials concluding that regular aerobic exercise during pregnancy (mostly swimming, static cycling, floor exercises) improves women's physical fitness, but that there was insufficient evidence to draw firm conclusions about any other short- or long-term risks or benefits to the woman or her offspring [1].

Physical activity is associated with lower cardiovascular risk in both adults and children [6-8]. It is possible that greater levels of physical activity during pregnancy might improve later offspring cardiovascular health, either through direct intrauterine mechanisms influencing musculoskeletal or other system development or epigenetic changes, or because mothers who are more active in pregnancy are also likely to be so postnatally and might encourage their children to be more active [9-12]. There is some evidence that greater levels of physical activity during pregnancy is associated with later offspring growth and lower adiposity [13-16]. All but one of these studies [16] had a very small sample size, and none were able to assess ~~other (than adiposity)~~ cardiovascular risk factors other than adiposity in offspring. Furthermore, a recent study [17] found evidence of an adverse effect of physical activity measured at 30 weeks gestation on high density lipoprotein cholesterol, diastolic blood pressure and body mass index (BMI). Thus, it is unclear whether variation in levels of physical activity during pregnancy is (either beneficially or detrimentally) related to long-term cardiovascular health in offspring.

The aim of this study is to investigate the association of physical activity during pregnancy with a range of offspring cardiovascular risk factors (BMI, waist circumference, glucose, insulin, lipids, and blood pressure). We further aim to explore whether any observed associations are explained by measured confounders, or if not, whether they are mediated by intrauterine characteristics such as hypertensive disorders of pregnancy (HDP), gestational diabetes or gestational weight gain, or whether they are mediated by the offspring's own later physical activity levels.

For peer review only - http://bmjopen.bmj.com/site/about/guidelines.xhtml

METHODS

The Avon Longitudinal Study of Parents and Children (ALSPAC) is a prospective, population-based study investigating environmental and other factors that affect the health and development of children. The study methods are described in detail elsewhere [18 19] and on the study website (**http://www.alspac.bris.ac.uk**). In brief, all pregnant women living in the three health districts centered around Bristol, in the South West of England, who had an expected delivery date between April 1, 1991 and December 31, 1992 were invited to take part in the study. Ethics approval for the study was obtained from the ALSPAC Law and Ethics Committee and the Local Research Ethics Committee.

From the 14,062 live births in the study, we restricted analyses to singleton pregnancies of offspring alive at 1 year, and excluded 1,234 women who did not provide data on physical activity during pregnancy, 1 woman with an implausible value of physical activity during pregnancy and 7,063 offspring without data for at least one outcome, yielding a sample size of 4,665 mother-offspring pairs (see Fig. 1~~Fig. 1~~ for the study participant flow diagram).

Physical activity during pregnancy

At 18 weeks gestation~~,~~ women were asked, in a validated questionnaire [20], to report the hours per week that they currently spend doing each of 11 types of leisure time physical activities (brisk walking, swimming, antenatal exercise, keep fit exercise, cycling, aerobics, tennis, yoga, jogging, weight training, squash, other exercise). Based on the responses to these questions, we estimated a weighted activity index (i.e. the weekly total energy expenditure from these activities) by multiplying the published average metabolic equivalent (MET) scores for the reported activities [21] with the estimated hours per week (7 for ‘‘≥7 h’’, 4 for ‘‘2–6 h’’, 0.5 for ‘‘<1 h’’, and 0 for ‘‘never’’) – see Supplemental Table 1 (Online Resource) for example MET scores. MET scores are defined as the ratio of the work metabolic rate to a standard resting metabolic rate of 1.0 kcal·kg^{-1}·hr^{-1} [22]. MET scores were derived from published sources that measured the oxygen cost of each activity, or estimated from the MET scores of similar activities where no studies could be found for this activity [22]. Published MET values are not available for ‘antenatal exercise’ and ‘keep-fit exercise’, thus we assigned 2.0 and 3.5 METs, respectively. Physical activity equating to a score greater than 70 was reported by 46 individuals, which is equivalent to 7 hours per week of jogging; these values were re-coded to 70 in order to prevent them from having undue influence on the results [22].

For peer review only

Cardiovascular risk factors in the offspring

Offspring cardiovascular risk factors (body mass index (BMI), waist circumference, glucose, insulin, lipids (high density lipoprotein cholesterol (HDLc) and low density lipoprotein cholesterol (LDLc)), and blood pressure) were measured at a research clinic attended at mean age 15.5 years (further details are provided in Online Resource). Weight and height were measured with the child in light clothing without shoes. BMI was calculated as weight in kilograms divided by height in metres squared. A flexible tape was used to measure waist circumference to the nearest 1 mm at the mid-point between the lower ribs and the pelvic bone. Blood pressure was measured with a Dinamap 9301 Vital Signs Monitor (Morton Medical, London) with the correct cuff size. Two readings of systolic and diastolic blood pressure were recorded, with the child at rest and their arm supported, and we used the mean of the two measures. A fasting blood sample was taken and used to measure total cholesterol, triglycerides and HDLc. LDLc concentrations were determined from these with the Friedwald equation (LDLc=total cholesterol−HDLc+triglycerides×0.45 mmol/l)). Insulin was measured with an enzyme linked immunosorbent assay (ELISA) (Mercodia, Uppsala, Sweden) that does not cross-react with proinsulin, and plasma glucose was measured with an automated assay. Further details are provided in Online Resource.

Other measurements

We considered the following to be potential confounding factors: household social class, maternal education, ethnicity, parity (the number of previous pregnancies resulting in a live birth or stillbirth), smoking during pregnancy, previous history of hypertension outside of pregnancy, pre-pregnancy BMI, and gestational weight gain in the first 18 weeks of pregnancy. We also adjusted for the offspring's gender and age at assessment of outcomes in all analyses.

We considered the following to be potential mediating factors: HDP (mothers with pre-eclampsia or gestational hypertension), gestational weight gain (between 18-28 weeks and 28 weeks onwards; see Online Resource for explanation of why these two time points were used), gestational diabetes, offspring gestational age, birthweight and offspring accelerometer-assessed moderate to vigorous physical activity at 14 years. Gestational age and birthweight are plausible mediators of the intrauterine pathways due to their association with both gestational diabetes and HDP, and offspring outcomes [10 11]. Details of the assessment of all of these potential confounders and mediators are provided in Online Resource.

For peer review only - http://bmjopen.bmj.com/site/about/guidelines.xhtml

Statistical Analysis

All analysis was performed with Stata v11.2 [23]. Insulin and triglycerides were positively skewed and so were log transformed. Multivariable linear regression was used to examine the association between the weighted score of maternal physical activity during pregnancy and offspring cardiovascular risk factors. The weighted score of maternal physical activity in pregnancy was standardized to have a mean of zero and variance of one. Coefficients from regression models therefore represent the mean difference in cardiovascular risk factors (in their measured units or a percentage difference for the log transformed insulin and triglyceride outcomes) for a one standard deviation increase in MET physical activity, which is equivalent to approximately 15 MET hours per week, i.e. just over two hours of jogging, or just under four hours of brisk walking. We also repeated all analyses with each cardiovascular outcome scaled per standard deviation of their distribution (or logged distribution for insulin and triglycerides) and present these results in figures. This allowed us to compare the magnitudes of any associations across outcomes. We used tests of interaction to assess whether the associations between maternal physical activity during pregnancy and offspring cardiovascular risk factors differed by child gender or by maternal pre-pregnancy BMI (categorized as underweight/normal weight [BMI less than 25] or overweight/obese [BMI 25 or above]) [24].

Fig. 2~~Fig. 2~~ illustrates the hypothesised pathways between maternal physical activity during pregnancy and offspring cardiovascular risk factors. We carried out several analyses to explore these pathways. We initially investigated minimally adjusted associations, adjusting only for the child's gender and exact age at outcome measurement. Confounder-adjusted models adjusted for child gender, exact age at outcome measurement, household social class, maternal education, ethnicity, parity, smoking during pregnancy, previous hypertension, pre-pregnancy weight and gestational weight gain between 0-18 weeks gestation. The possible mediating mechanisms involved were investigated in two further models: a) all confounders plus intrauterine characteristics – HDP, gestational diabetes and gestational weight gain after 18 weeks, gestational age and birthweight, and b) all confounders plus offspring physical activity (measured objectively using an accelerometer) at age 14. A final model included all potential confounding and mediating factors.

Missing data

We used multivariate multiple imputation (details in Online Resource) to impute missing data for all mother-offspring pairs where the data on maternal physical activity during pregnancy (the exposure) and at least one cardiovascular risk factor at age 15 was observed (the eligible sample, N=4,665). Analyses conducted using this

For peer review only - http://bmjopen.bmj.com/site/about/guidelines.xhtml

imputation dataset were considered our main analyses. However, in order to assess the impact of missing data on our results, we compared the results of the main analysis (using imputed datasets) with results from a complete case approach in which we included only individuals with complete data for all outcomes, all confounders and all mediators. Characteristics of participants and levels of missing data for each variable are shown in Supplementary Table 2 (Online Resource).

For peer review only

For peer review only - http://bmjopen.bmj.com/site/about/guidelines.xhtml

RESULTS

There was large inter-individual variation in reported MET-hours per week at 18 weeks gestation. Approximately one third of mothers reported less than 6 MET hours per week, which equates to one hour of swimming, whilst approximately one quarter of women reported over 24 MET hours per week. On average, higher social class, greater maternal education, lower parity, no maternal smoking in pregnancy and lower maternal pre-pregnancy BMI were associated with greater reported physical activity at 18 weeks gestation (Table 1). There was limited evidence that maternal physical activity was associated with most of our a priori specified mediators of the association between physical activity in pregnancy and offspring cardiovascular risk factors; no strong evidence of association was seen for hypertensive disorders of pregnancy, diabetes, gestational weight gain, gestational age, or birth weight, but greater maternal physical activity during pregnancy was associated with greater offspring physical activity at age 14. For each standard deviation increase in reported physical activity during pregnancy, there was a 0.06 SD increase in physical activity in the offspring (95% CI 0.03 to 0.09) (Supplementary Table 3 in Online Resource).

Association between physical activity during pregnancy and offspring cardiovascular risk factors

There was no evidence that the associations between maternal physical activity during pregnancy and offspring cardiovascular risk factors differed either by offspring gender (all *P* for interaction > 0.2) or by maternal pre-pregnancy BMI status (all *P* for interaction > 0.1 see Supplemental Figure 1 in Online Resource). Fig. 3Fig. 3 and Table 2 show the minimally adjusted, confounder adjusted and mediator (intrauterine characteristics and offspring physical activity) adjusted associations of maternal physical activity during pregnancy with offspring cardiovascular risk factors. More detailed results from models examining each potential mediator separately are given in Supplemental Table 4 in Online Resource).

There was little evidence that maternal physical activity during pregnancy was associated with the offspring cardiovascular risk factors. In minimally adjusted analyses (adjusting only for child gender and age at outcome assessment), higher levels of physical activity were associated with lower offspring BMI, smaller waist circumference, lower glucose, and lower insulin. For all outcomes except insulin, and glucose, BMI and waist circumference, the magnitude of coefficients was small and confidence intervals were wide. A one standard deviation increase in physical activity during pregnancy is associated with a lower insulin level in the offspring of -1.90% (95% CI -3.50 to -0.30) and lower glucose level by -0.013 mmol/l (95% CI -0.027 to 0.001).

For peer review only - http://bmjopen.bmj.com/site/about/guidelines.xhtml

Adjustment for measured confounders moved most of the associations towards the null. After adjusting for confounding, one standard deviation increase in physical activity during pregnancy is associated with lower offspring insulin by -1.20% (95% CI -2.80 to 0.40). The borderline association with glucose did not alter after adjusting for confounders. Adjusting for potential mediators had little effect on the estimated associations for any outcome.

Sensitivity of results to missing data

The results based on multivariate multiple imputation were very similar to those based on individuals with complete data (Supplemental Figure 2 for BMI, Supplemental Figure 3 for insulin and Supplemental Figure 4 for glucose are shown as illustrative examples, in Online Resource).

For peer review only - http://bmjopen.bmj.com/site/about/guidelines.xhtml

DISCUSSION

Despite greater maternal physical activity in pregnancy being associated with lower pre-pregnancy BMI and greater offspring physical activity at mean age 14 years, there was no strong evidence that it was related to later offspring cardiovascular risk factors (BMI, waist circumference, blood pressure (systolic blood pressure, diastolic blood pressure), lipids (triglycerides, low density lipoprotein cholesterol, high density lipoprotein cholesterol), and fasting glucose or insulin) at age 15 years using data from a contemporary general population pregnancy cohort. The associations we observed were small, and were generally attenuated with adjustment for measured confounders. The strongest evidence of association was for glucose (one standard deviation greater physical activity in pregnancy was associated with a lower glucose by -0.013 mmol/l (95% confidence interval -0.027 to 0.001), equivalent to approximately one third of a standard deviation), but given that we investigated several outcomes, that the association with all other outcomes including insulin attenuated towards the null after adjustment for confounding, and that no associations were seen for BMI, waist circumference or any other cardiovascular risk factor, this finding should be interpreted with caution and requires replication in other studies.

To our knowledge only one study to date has investigated the association of maternal physical activity during pregnancy with a range of offspring cardiovascular risk factors [17]. This study included 965 Danish participants at age 20, and examined the associations of self-reported physical activity at work, leisure time physical activity, daily amount of walking or cycling, and sport participation between 18 and ~~at~~ 30 weeks gestation (measured at 30 weeks gestation), with offspring cardiovascular risk factors. The authors found that higher levels of self-reported walking-cycling at 30 weeks gestation was associated with higher offspring BMI and diastolic blood pressure, lower high density lipoprotein cholesterol, and (in males only) higher systolic blood pressure and waist circumference. The associations were weak and of small magnitude. No associations were seen for the other aspects of physical activity assessed in the analysis. It is therefore possible that the associations observed in this study occurred due to chance. However, the authors' conclusions are in line with our own, in that there was no evidence to support the a priori hypothesised potential benefit of physical activity during pregnancy for offspring cardiovascular health.

The main strengths of our study are the large sample size, prospective nature of data collection, and the detailed measurement of a wide range of offspring cardiovascular risk factors in adolescence. The measure of maternal physical activity during pregnancy uses a weighted index, which attempts to measure the energy

For peer review only - http://bmjopen.bmj.com/site/about/guidelines.xhtml

expenditure of individuals using previously published average values for different types of physical activity (see Supplemental Table 1 in Online Resource). We were able to assess the extent to which our observed associations are confounded by maternal pre-pregnancy BMI and socio-economic factors, and explore a number of potential a priori specified mediating pathways (intrauterine factors and shared familial environment, represented by the child's own physical activity). Additionally, we used multivariate multiple imputation to examine and correct for potential bias introduced by missing data.

The results using complete case data and using multivariate multiply imputed data were similar. In both analyses, the conclusion remains that there is no strong association between reported levels of physical activity during pregnancy and offspring BMI (or any other cardiovascular risk factor). Multiple imputation gives unbiased results providing the data are missing at random, i.e. assuming that the missingness is not related to the value of the observation, conditional on the variables included in the imputation model. We included in the imputation models a wide range of variables predictive of both missingness in the outcomes and of levels of cardiovascular risk factors in those with observed data, including socio-economic factors, maternal and family characteristics, and earlier measures of the offspring's cardiovascular disease risk factors, in order to make sure that this assumption holds. The loss to follow-up in our study would result in selection bias if the association between maternal physical activity during pregnancy and offspring cardiovascular risk differed between those included in our analyses and those lost to follow-up. However, various studies have investigated the potential effects of loss to follow-up on associations in epidemiological studies, and have found that they are generally robust to even severe loss to follow-up [25-28].

A limitation of our work is that the data on maternal physical activity during pregnancy were self-reported, and thus measurement error is ~~possible~~inevitable. It is possible that the women may under- or over-report their physical activity levels but we do not have objective data with which to evaluate this for our participants [20]. However, associations of socioeconomic position, pre-pregnancy BMI, maternal education and objectively measured offspring physical activity at age 14 were as expected, providing some evidence of face validity. MET scores may not accurately reflect the energy expenditure of physical activity in pregnancy, as they are not derived from studies of pregnant individuals. However, this is inconsequential as we use this measure as an activity classification scheme rather than an exact quantification of energy expenditure [29], and MET scores have been used in this way in several previous studies [30 31]. The "other" activity category was selected by 9.45% of the women in our study and it is possible this may reduce the strength of the associations, as several activity types with different energy expenditures are assigned the same MET score. We cannot assess women's

For peer review only - http://bmjopen.bmj.com/site/about/guidelines.xhtml

physical activity levels before pregnancy, or the extent to which women changed their physical activity levels during pregnancy, both before and after between the 18 week questionnaire, and the end of the pregnancy as these data are not availablethis measure of physical activity was only captured once in this cohort. Previous research with this cohort has shown that two thirds of women report at 18 weeks gestation that they have reduced their physical activity levels since becoming pregnant, but at 18 and 32 weeks gestation similar proportions of women report at least 3 hours per week of strenuous physical activity (defined as 'sufficient to cause sweating') [22]. Thus it is possible that physical activity either earlier or later in pregnancy may be a more important exposure for offspring cardiovascular risk – further studies are necessary to test this hypothesis.

Although we demonstrated no evidence of association between physical activity during pregnancy and offspring cardiovascular risk factors, we did find that greater levels of physical activity during pregnancy were associated with greater levels of objectively assessed physical activity in the offspring at mean age 14 years. This measure of offspring physical activity has been shown to be associated with fat mass and cardiovascular risk factors [8 32].

Our results, in line with the only existing similar study to date [17], suggest that maternal physical activity in pregnancy is unlikely to be an important determinant of later offspring cardiovascular health, though further large prospective studies of this association are needed to be confident that this is the case, particularly with studies using objectively measured physical activity at various times throughout pregnancy.

For peer review only

For peer review only - http://bmjopen.bmj.com/site/about/guidelines.xhtml

FOOTNOTES

Contributors

Louise A C Millard: Ms. Millard contributed to the design of the study, performed the analyses, drafted the initial manuscript, reviewed and revised the manuscript, and approved the final manuscript as submitted. Debbie A Lawlor: Professor Lawlor conceptualised and designed the study, critically reviewed and revised the manuscript, and approved the final manuscript as submitted. Abigail Fraser: Dr Fraser contributed to the design of the study, critically reviewed and revised the manuscript, and approved the final manuscript as submitted. Laura D Howe: Dr Howe contributed to the design of the study, supervised the analyses, critically reviewed and revised the manuscript, and approved the final manuscript as submitted.

Funding

LACM is funded by a studentship from the UK Medical Research Council. LDH was funded by a UK Medical Research Council Population Health Scientist Fellowship (G1002375). AF is funded by a UK Medical Research Council research fellowship (grant 0701594). The UK Medical Research Council (grant G074882), the Wellcome Trust (grant WT076467), and the University of Bristol provide core funding support for ALSPAC. The UK Medical Research Council (grant G0600705) and the University of Bristol provide core funding for the Medical Research Council Centre for Causal Analyses in Translational Epidemiology.

Ethics approval

ALSPAC Law and Ethics Committee and the Local Research Ethics Committees.

Data sharing statement

The ALSPAC policy on data sharing is available on the website: http://www.bristol.ac.uk/alspac. To discuss access to ALSPAC data, please contact the ALSPAC executive team on alspac-exec@bristol.ac.uk.

For peer review only - http://bmjopen.bmj.com/site/about/guidelines.xhtml

TABLES

Table 1 Association of participant characteristics with maternal physical activity during pregnancy: using multivariate multiply imputed data (N = 4,665) of individuals with data for maternal physical activity (exposure) and at least one outcome

Potential confounder	Confounder category	Standard deviation (SD) change of maternal physical activity during pregnancy associated with a 1 SD increase of confounder, with 95% confidence interval
Household social class [a]	Class I (professional)	Reference
	Class II	0.00 (-0.08 to 0.09)
	Class III non-manual	-0.18 (-0.27 to -0.09)
	Class III manual	-0.16 (-0.28 to -0.04)
	Class IV/V (manual)	-0.15 (-0.31 to 0.01)
Maternal education [b]	Less than O-level	Reference
	O-level	0.17 (0.09 to 0.25)
	A-level	0.39 (0.30 to 0.47)
	Degree or above	0.40 (0.30 to 0.49)
Ethnicity [c]	Non-white	Reference
	White	-0.11 (-0.25 to 0.03)
Parity [d]	0	Reference
	1	-0.05 (-0.11 to 0.02)
	2 +	-0.10 (-0.19 to -0.02)
Maternal smoking in pregnancy	No	Reference
	Yes	-0.09 (-0.17 to -0.01)
Previous hypertension	No	Reference
	Yes	-0.03 (-0.17 to 0.12)
Maternal pre-pregnancy BMI (SD)		-0.39 (-0.58 to -0.20)
Gestational weight gain 0-18 weeks (SD)		-0.00 (-0.03 to 0.03)

[a] Household social class: The mother recorded the occupation of both herself and her partner in a questionnaire at 32 weeks gestation, which were used to allocate them to social class groups using the 1991 Office of Population, Censuses and Surveys classification; the highest class of the mother and her partner was used in analysis
[b] Maternal education: O-level (ordinary level) exams are taken in different subjects usually at age 15–16 at the completion of legally required school attendance, equivalent to today's General Certificate of Secondary Education); A-level (Advanced-level) exams are taken in different subjects usually at age 18)
[c] Ethnicity: Mixed was recoded as non-white due to the small numbers in the mixed category (n=3)
[d] Parity: Obtained from obstetric records. Mothers with parity of two or more were grouped into a single category

For peer review only - http://bmjopen.bmj.com/site/about/guidelines.xhtml

Table 2 Association between maternal physical activity during pregnancy and offspring cardiovascular risk factors: using multivariate multiply imputed data (N = 4,665) of individuals with data for maternal physical activity (exposure) and at least one outcome

	Change per 1 SD greater maternal metabolic equivalent physical activity (95% confidence intervals)		
**Outcome**	**Minimally adjusted**	**Confounder adjusted**	**All confounders and mediators**
**Mean difference (in units specified in first column) per 1 standard deviation (SD) greater maternal physical activity during pregnancy**			
BMI, kg/m^2	-0.089 (-0.190 to 0.011)	0.004 (-0.091 to 0.098)	0.023 (-0.071 to 0.117)
Waist circumference, cm	-0.271 (-0.552 to 0.011)	-0.088 (-0.358 to 0.181)	-0.042 (-0.311 to 0.227)
SBP, mmHg	-0.044 (-0.360 to 0.272)	-0.014 (-0.332 to 0.303)	0.017 (-0.301 to 0.334)
DBP, mmHg	0.015 (-0.251 to 0.280)	0.017 (-0.251 to 0.284)	0.018 (-0.250 to 0.286)
Glucose, mmol/L	-0.013 (-0.027 to 0.001)	-0.013 (-0.027 to 0.001)	-0.012 (-0.026 to 0.001)
LDLc, mmol/L	-0.006 (-0.025 to 0.013)	-0.002 (-0.022 to 0.017)	-0.003 (-0.022 to 0.017)
HDLc, mmol/L	0.002 (-0.008 to 0.012)	0.000 (-0.010 to 0.010)	-0.001 (-0.011 to 0.009)
**Percentage difference per 1 standard deviation (SD) greater maternal physical activity during pregnancy**			
Insulin, % difference	-1.90 (-3.50 to -0.30)	-1.20 (-2.80 to 0.40)	-1.00 (-2.60 to 0.60)
Triglycerides, % difference	0.00 (-1.20 to 1.30)	0.00 (-1.30 to 1.20)	0.00 (-1.20 to 1.40)

Results of additional models with adjustment for mediators are in Supplemental Table 4 in Online Resource

Minimally adjusted: Adjusted for child gender and child age at 15 year clinic

Confounder adjusted: Adjusted for child age at 15 year clinic, child gender, maternal and family confounders ~~socioeconomic position confounders~~ (household social class, maternal education, ethnicity, parity, mother smoker, previous hypertension~~)~~, maternal pre-pregnancy BMI (weight & height) and gestational weight gain 0-18 weeks)

All confounders and mediators: Confounder adjusted with additional adjustment for intrauterine mechanisms (HDP (none, gestational hypertension, preeclampsia), diabetes/glycosuria (non, existing diabetes, gestational diabetes, glycosuria), gestational weight gain 18-24 weeks, gestational weight gain 24 weeks onwards), birth weight, gestational age, and child's physical activity at age 14

For peer review only - http://bmjopen.bmj.com/site/about/guidelines.xhtml

FIGURES

Fig. 1 Flow chart of ALSPAC study participants

Fig. 2 Pathway illustration for the potential associations between physical activity during pregnancy and offspring cardiovascular risk factors

Fig. 3 Associations of maternal physical activity during pregnancy with offspring cardiovascular risk factors: using multivariate multiply imputed data (N = 4,665) of individuals with data for maternal physical activity (exposure) and at least one outcome

Footnotes for Figure 3:

Mean standard deviation (SD) difference (and 95% confidence intervals) in offspring cardiovascular risk factors (or logged distribution for insulin and triglycerides) for a one SD increase in metabolic equivalent maternal physical activity

Minimally adjusted: Adjusted for child gender and child age at 15 year clinic

Confounder adjusted: Adjusted for child age at 15 year clinic, child gender, socio-economic position confounders (household social class, maternal education, ethnicity, parity, mother smoker, previous hypertension), maternal pre-pregnancy BMI (weight & height) and gestational weight gain 0-18 weeks

Fully adjusted with adjustment for all confounders and mediators: Confounder adjusted with additional adjustment for intrauterine mechanisms (HDP (none, gestational hypertension, preeclampsia), diabetes/glycosuria (non, existing diabetes, gestational diabetes, glycosuria), gestational weight gain 18-24 weeks, gestational weight gain 24 weeks onwards), birth weight, gestational age, and child's physical activity at age 14

For peer review only - http://bmjopen.bmj.com/site/about/guidelines.xhtml

REFERENCES

1. Kramer MS, McDonald SW. Aerobic exercise for women during pregnancy. Cochrane Database Syst Rev 2006;**3**:CD000180 doi: 10.1002/14651858.CD000180.pub2[published Online First: Epub Date]|.
2. ACOG Committee opinion. Number 267, January 2002: exercise during pregnancy and the postpartum period. Obstet Gynecol 2002;**99**(1):171-3 doi: S0029784401017495 [pii][published Online First: Epub Date]|.
3. Davies GA, Wolfe LA, Mottola MF, MacKinnon C. Joint SOGC/CSEP clinical practice guideline: exercise in pregnancy and the postpartum period. Can J Appl Physiol 2003;**28**(3):330-41
4. (RCOG) RCoOaG. Exercise in pregnancy. Secondary Exercise in pregnancy [HTML] January 2006 2006. http://www.rcog.org.uk/womens-health/clinical-guidance/exercise-pregnancy.
5. Physical activity guidelines for Americans. Okla Nurse 2008;**53**(4):25
6. Haskell WL, Lee IM, Pate RR, et al. Physical activity and public health: updated recommendation for adults from the American College of Sports Medicine and the American Heart Association. Circulation 2007;**116**(9):1081-93 doi: CIRCULATIONAHA.107.185649 [pii]

10.1161/CIRCULATIONAHA.107.185649[published Online First: Epub Date]|.
7. Sallis JF, Patterson TL, Buono MJ, Nader PR. Relation of cardiovascular fitness and physical activity to cardiovascular disease risk factors in children and adults. Am J Epidemiol 1988;**127**(5):933-41
8. Ekelund U, Luan JA, Sherar LB, et al. Moderate to Vigorous Physical Activity and Sedentary Time and Cardiometabolic Risk Factors in Children and Adolescents. Jama-J Am Med Assoc 2012;**307**(7):704-12
9. Jayet PY, Rimoldi SF, Stuber T, et al. Pulmonary and systemic vascular dysfunction in young offspring of mothers with preeclampsia. Circulation 2010;**122**(5):488-94
10. Lawlor DA, Macdonald-Wallis C, Fraser A, et al. Cardiovascular biomarkers and vascular function during childhood in the offspring of mothers with hypertensive disorders of pregnancy: findings from the Avon Longitudinal Study of Parents and Children. Eur Heart J 2011 doi: 10.1093/eurheartj/ehr300[published Online First: Epub Date]|.
11. Lawlor DA, Fraser A, Lindsay RS, et al. Association of existing diabetes, gestational diabetes and glycosuria in pregnancy with macrosomia and offspring body mass index, waist and fat mass in later childhood: findings from a prospective pregnancy cohort. Diabetologia 2010;**53**(1):89-97 doi: 10.1007/s00125-009-1560-z[published Online First: Epub Date]|.
12. Dabelea D, Hanson RL, Lindsay RS, et al. Intrauterine exposure to diabetes conveys risks for type 2 diabetes and obesity: a study of discordant sibships. Diabetes 2000;**49**(12):2208-11
13. Clapp JF, 3rd. Morphometric and neurodevelopmental outcome at age five years of the offspring of women who continued to exercise regularly throughout pregnancy. The Journal of pediatrics 1996;**129**(6):856-63
14. Clapp JF, 3rd, Simonian S, Lopez B, Appleby-Wineberg S, Harcar-Sevcik R. The one-year morphometric and neurodevelopmental outcome of the offspring of women who continued to exercise regularly throughout pregnancy. American journal of obstetrics and gynecology 1998;**178**(3):594-9
15. Pomeroy J, Renstr√∂m F, Gradmark AM, et al. Maternal Physical Activity and Insulin Action in Pregnancy and Their Relationships With Infant Body Composition. Diabetes Care 2012 doi: 10.2337/dc12-0885[published Online First: Epub Date]|.
16. Camilla Schou Andersen MJ, Michael Gamborg, Thorkild I. A. Sørensen, and Ellen Aagaard Nohr. Maternal Recreational Exercise during Pregnancy in relation to Children's BMI at 7 Years of Age. International Journal of Pediatrics 2012;**2012**:8 doi: 10.1155/2012/920583[published Online First: Epub Date]|.
17. Danielsen I, Granstrom C, Rytter D, et al. Does physical activity during pregnancy adversely influence markers of the metabolic syndrome in adult offspring? A prospective study over two decades. J Epidemiol Community Health 2013;**67**(8):648-54 doi: 10.1136/jech-2012-202017

For peer review only - http://bmjopen.bmj.com/site/about/guidelines.xhtml

jech-2012-202017 [pii][published Online First: Epub Date]|.
18. Boyd A, Golding J, Macleod J, et al. Cohort Profile: the 'children of the 90s'--the index offspring of the Avon Longitudinal Study of Parents and Children. International journal of epidemiology 2013;**42**(1):111-27 doi: 10.1093/ije/dys064

dys064 [pii][published Online First: Epub Date]|.
19. Fraser A, Macdonald-Wallis C, Tilling K, et al. Cohort Profile: the Avon Longitudinal Study of Parents and Children: ALSPAC mothers cohort. International journal of epidemiology 2013;**42**(1):97-110 doi: 10.1093/ije/dys066

dys066 [pii][published Online First: Epub Date]|.
20. Wildschut HI, Harker LM, Riddoch CJ. The potential value of a short self-completion questionnaire for the assessment of habitual physical activity in pregnancy. J Psychosom Obstet Gynaecol 1993;**14**(1):17-29
21. Ainsworth BE, Haskell WL, SD H, et al. Compendium of Physical Activities: a second update of codes and MET values. Med Sci Sports Exerc 2011
22. Liu J, Blair SN, Teng Y, Ness AR, Lawlor DA, Riddoch C. Physical activity during pregnancy in a prospective cohort of British women: results from the Avon longitudinal study of parents and children. European journal of epidemiology 2011;**26**(3):237-47 doi: 10.1007/s10654-010-9538-1[published Online First: Epub Date]|.
23. Stata Statistical Software: Release 11 [program], 2009.
24. Clinical Guidelines on the Identification, Evaluation, and Treatment of Overweight and Obesity in Adults--The Evidence Report. National Institutes of Health. Obes Res 1998;**6 Suppl 2**:51S-209S
25. Howe LD, Tilling K, Galobardes B, Lawlor DA. Loss to follow-up in cohort studies: bias in estimates of socioeconomic inequalities. Epidemiology 2013;**24**(1):1-9 doi: 10.1097/EDE.0b013e31827623b1[published Online First: Epub Date]|.
26. Nilsen RM, Vollset SE, Gjessing HK, et al. Self-selection and bias in a large prospective pregnancy cohort in Norway. Paediatr Perinat Epidemiol 2009;**23**(6):597-608 doi: 10.1111/j.1365-3016.2009.01062.x

PPE1062 [pii][published Online First: Epub Date]|.
27. Nohr EA, Frydenberg M, Henriksen TB, Olsen J. Does low participation in cohort studies induce bias? Epidemiology 2006;**17**(4):413-18
28. Pizzi C, De Stavola BL, Pearce N, et al. Selection bias and patterns of confounding in cohort studies: the case of the NINFEA web-based birth cohort. J Epidemiol Community Health 2012;**66**(11):976-81 doi: 10.1136/jech-2011-200065

jech-2011-200065 [pii][published Online First: Epub Date]|.
29. Ainsworth BE, Haskell WL, Whitt MC, et al. Compendium of physical activities: an update of activity codes and MET intensities. Med Sci Sports Exerc 2000;**32**(9 Suppl):S498-504
30. Borodulin KM, Evenson KR, Wen F, Herring AH, Benson AM. Physical activity patterns during pregnancy. Med Sci Sports Exerc 2008;**40**(11):1901-8 doi: 10.1249/MSS.0b013e31817f1957[published Online First: Epub Date]|.
31. Rousham EK, Clarke PE, Gross H. Significant changes in physical activity among pregnant women in the UK as assessed by accelerometry and self-reported activity. Eur J Clin Nutr 2006;**60**(3):393-400
32. Riddoch CJ, Leary SD, Ness AR, et al. Prospective associations between objective measures of physical activity and fat mass in 12-14 year old children: the Avon Longitudinal Study of Parents and Children (ALSPAC). Brit Med J 2009;**339** doi: ARTN b4544

DOI 10.1136/bmj.b4544[published Online First: Epub Date]|.

For peer review only - http://bmjopen.bmj.com/site/about/guidelines.xhtml
